# Co-Design of a Depression Self-Management Tool for Adolescent and Young Adult Cancer Survivors: Rapid Qualitative Analysis of Interview Feedback on a Prototype

**DOI:** 10.2196/77994

**Published:** 2026-04-13

**Authors:** Karly M Ingram, Grace Westcott, Antonija Augustinovic, Rachel Glock, AnneMarie Coffey, David Victorson, DerShung Yang, Madhu Reddy, Sarah A Birken, John M Salsman

**Affiliations:** 1 Department of Psychology Thomas Harriot College of Arts & Sciences East Carolina University Greenville, NC United States; 2 Department of Psychology Virginia Commonwealth University Richmond United States; 3 Department of Medical Social Sciences Feinberg School of Medicine Northwestern University Chicago, IL United States; 4 BrightOutcome (United States) Buffalo Grove United States; 5 Department of Informatics University of California, Irvine Irvine United States; 6 Department of Implementation Science School of Medicine Wake Forest University Winston-Salem United States; 7 Department of Social Sciences and Health Policy School of Medicine Wake Forest University Winston-Salem, NC United States

**Keywords:** adolescent and young adult, cancer survivorship, depressive symptoms, self-management, individual interviews, user-centered design, rapid qualitative analysis, intervention tailoring, digital mental health

## Abstract

**Background:**

Over 2.1 million adolescent and young adult cancer survivors (AYACS) live in the United States. Recent estimates suggest that up to one-third of AYACS experience major depressive disorder. Although several efficacious evidence-based interventions are available to manage symptoms of depression, these interventions are often inaccessible to AYACS who have many competing commitments. Digital mental health tools hold promise for this population; however, only a few have been tailored to meet the unique needs of AYACS, and findings to date have yielded mixed results.

**Objective:**

This study aims to obtain feedback from AYACS on a mid-fidelity prototype of a depression self-management tool being tailored for AYACS.

**Methods:**

Individuals with a history of cancer diagnosed at age 12 or older who were between the ages of 15 and 39 and had completed primary treatment were identified through a review of medical records from a comprehensive cancer center in the Southeastern United States. Potentially eligible participants were contacted by study staff to conduct additional screening and obtain informed consent via REDCap (Research Electronic Data Capture; Vanderbilt University). Upon enrollment, participants provided demographic and clinical information, as well as their availability for an interview. The principal investigator (KMI) conducted semistructured individual interviews with consented AYACS. Most of the interview was dedicated to showing participants the mid-fidelity prototype of the tool, explaining how the prototype might work, and requesting targeted feedback. Demographic and clinical characteristics, as well as some aspects of feedback on the prototype, were summarized using descriptive statistics. Interviews were audio- and video-recorded and transcribed. The transcriptions underwent rapid qualitative analysis guided by the Rigorous and Accelerated Data Reduction technique.

**Results:**

A total of 14 AYACS (n=9, 64%, female; n=9, 64%, white; ages 15-38) completed an individual interview. Participant preferences for mood tracking, content presentation, user input, and duration of use were captured qualitatively but analyzed quantitatively. For example, most participants (n=10, 71%) indicated that they preferred a mood-tracking option that included emojis and would be willing to track their mood at least once per day (n=11, 79%). Participant preferences captured qualitatively fell into 4 themes: (1) features to promote user engagement (eg, the use of gamification); (2) tailored content presentation (eg, authenticity in the portrayal of the cancer experience); (3) perceived usability (eg, simplifying user input); and (4) interface design (eg, implementing a coherent design theme and color scheme).

**Conclusions:**

Findings indicated that AYACS highly value personalization, flexibility, and peer support in digital interventions. Based on insights obtained during individual interviews, a working prototype was developed by reprogramming an existing digital tool. Qualitative and quantitative findings informed modifications to the existing digital tool. The working prototype will next undergo evaluation as part of a pilot full-factorial trial.

## Introduction

As of 2020, around 2.1 million adolescent and young adult (AYA; ages 15-39) cancer survivors (AYACS) live in the United States, with this number projected to grow as the incidence of cancer in this age group rises. Additionally, nearly 90,000 AYAs receive a cancer diagnosis each year in the United States [1,2]. Given the complexities of an AYACS’ life, significant disruptions to personal milestones (eg, career establishment, family development, and financial stability) may cause elevated depressive symptoms [3].

Indeed, it is estimated that AYACS are 1.3-fold more likely to experience depression than older cancer survivors or cancer-free counterparts [4]. Increased depressive symptoms can result in poorer quality of life, lower adherence to medical recommendations, increased health care costs, and elevated mortality risks [5]. Unlike other factors that contribute to these problematic outcomes (eg, genetics), symptoms of depression are treatable and represent an ideal target for behavioral interventions. AYACS are estimated to have a prevalence of major depressive disorder of 25%-32% [6].

The American Psychological Association clinical practice guidelines recommend cognitive behavioral therapy (CBT) as an effective method for treating depressive symptoms [7]. CBT is well documented as an effective treatment for managing depressive symptoms in patients with cancer and cancer survivors [8-10]. Patients who experience depressive symptoms can utilize CBT to identify and alter negative automatic thoughts and maladaptive behaviors through the practice of cognitive restructuring and behavioral activation [11]. Expanding on CBT, mindfulness-based CBT integrates principles of mindfulness-based stress reduction, which encourages individuals to develop a different relationship with their thoughts and emotions [7,12]. This added focus on mindfulness may further enhance the effectiveness of treating depression by promoting greater acceptance and awareness of one’s mental state. In addition to these evidence-based therapies, a growing body of literature highlights the use of positive psychology interventions to improve mental health outcomes in individuals with severe and chronic illnesses. Recent systematic reviews and meta-analyses of positive psychology interventions found a small-to-moderate effect on depressive symptoms and suggest that these interventions may be more effective than other interventions targeting depression [13,14]. Overall, several efficacious evidence-based interventions (EBIs) are available to manage symptoms of depression. AYACS face a heightened risk of depression, yet the accessibility of EBIs for managing depression remains uncertain. Findings from the AYA Health Outcomes & Patient Experience study revealed that 56%-75% of AYACS reported unmet psychosocial support needs 1 year after diagnosis [15].

Providing accessible psychosocial interventions to support the AYACS population is critical for managing and treating depressive symptoms [16]. Barriers to accessing psychosocial interventions for AYACS are limited access to in-person services and difficulties fitting therapy sessions into their schedules, as many are offered only during standard business hours [16]. Digital mental health interventions offer numerous benefits that enhance the accessibility of mental health care for busy AYACS. Digital tools provide a convenient way to access support and resources, allowing individuals to seek help from the comfort of their homes. Furthermore, digital mental health solutions can reach underserved populations in remote or low-income areas [17]. EBIs can be provided remotely through smartphone technology, which is widely accessible to most AYACS, enabling broad scalability [18,19]. Research has shown that digital tools can effectively support AYACS in managing physical symptoms [20]. Additionally, psychosocial interventions have been successfully adapted for digital formats, demonstrating their effectiveness in healthy AYA populations [21-24]. Despite this progress, there is limited research assessing the impact of digital interventions that specifically assist AYACS with depression. For instance, a recent meta-analysis of online mental health interventions targeting pediatric and AYACS revealed a small pooled effect size (*g*=0.17, 95% CI –0.40 to 0.74) with significant heterogeneity (*I*^2^=71%) and no significant differences between intervention and control groups (*Z*=0.59, *P*=.55) [25]. This finding highlights the overall limited and inconsistent efficacy of current interventions for managing depressive symptoms in AYACS. As such, there is a critical need to develop efficacious interventions to manage depressive symptoms in AYACS.

This study is part of a larger, 5-year project that follows the Multiphase Optimization Strategy framework [26]. The framework comprises 3 distinct phases. The preparation phase sets the groundwork for the intervention’s development, involving the selection of a guiding conceptual model, the identification of potential intervention components, and the completion of formative and pilot research. The optimization phase uses a factorial experiment to assess which components most effectively meet the criteria for improving the intervention [26,27]. Lastly, the evaluation phase tests the efficacy of the optimized intervention. Our work to date addresses tasks within the preparation phase, gathering formative data that will shape the design and functionality of the digital tool [28]. This work has been informed by the user-centered design process proposed by McCurdie et al [29]. This process begins with concept generation and ideation involving the user. To achieve this step, we previously conducted co-design workshops to tailor a digital self-management tool for AYACS experiencing depressive symptoms [30]. We then engaged in initial prototype design, the second step of the user-centered design process. The resulting mid-fidelity prototype is a mock-up of the tool, which is planned to include features such as mood tracking, psychoeducation about depression and cancer, and adaptations of evidence-based care (eg, behavioral activation, cognitive restructuring, mindfulness training, and positive psychology). In this study, we engaged in the evaluation step of this process by conducting individual interviews with AYACS to collect targeted feedback on the mid-fidelity prototype. Finally, we returned to the second step of the user-centered design process by designing and developing a working prototype.

## Methods

### Overview

AYACS provided feedback on the mid-fidelity prototype during individual interviews. Individual interviews were conducted via Cisco Webex by KMI. KMI is a female researcher with a PhD in Clinical Psychology and extensive training in qualitative data collection methods, including a certificate in user-centered design. At the time of the study, KMI was an Instructor at the Wake Forest University School of Medicine. She did not have a relationship with study participants before study commencement, although some participants had previously engaged in co-design workshops with KMI as part of the larger study. Participants were informed that the primary purpose of the interviews was to inform the development of a digital tool to help AYACS better manage symptoms of depression. Each interview lasted approximately 1 hour. Transcripts of the individual interviews underwent rapid qualitative analysis to determine what changes should be made to the mid-fidelity prototype. The findings informed the design of a working prototype.

### Recruitment and Enrollment

Potentially eligible AYACS were identified through electronic medical record review at a large academic medical center in the Southeastern United States with a National Cancer Institute–designated comprehensive cancer center. At the time this study was conducted, this center served over 1300 AYACS annually. Based on the surrounding county demographics, the average minority distribution of AYACS was estimated to be approximately 13% African American, 2% Asian/Pacific Islander, 13% other/non-White, and 8% Hispanic/Latinx.

Potentially eligible participants were identified from the electronic medical record if they met the following criteria: (1) currently between the ages of 15 and 39; (2) diagnosed with cancer between 12 and 39 years of age; and (3) between 1 month and 5 years after completion of primary treatment for their cancer. Study staff contacted potentially eligible patients or their parents/guardians (for adolescents) by sending a message about the study through the electronic patient portal. Follow-up calls or emails were sent within 2 weeks to nonresponders. Across all modes of contact, we attempted to reach potentially eligible participants no more than 3 times before receiving a response. Recruitment materials directed interested individuals or their parents/guardians to a REDCap (Research Electronic Data Capture; Vanderbilt University) [31] screening form to confirm their eligibility for the study. In addition to evaluating the criteria described above, this form was also used to ensure that the patient (1) was fluent in spoken and written English; (2) owned a smartphone with a data plan; (3) had access to a computer with internet access, a webcam, and a microphone that could be used to participate in the interview; and (4) did not have a current diagnosis of a severe or persistent mental illness. Eligible patients and, in the case of adolescents, their parents/guardians were automatically directed to complete digital assent/consent forms. If eligibility could not be determined based on the screening form alone, study staff contacted the participant to make a final determination regarding eligibility for the study and, if eligible, manually sent the participant the digital assent/consent forms.

Upon providing assent/consent, AYACS were asked to provide demographic and clinical information, along with their availability to participate in study procedures. Study staff then contacted the participant to schedule the interview.

### Ethical Considerations

All study procedures were reviewed and approved by the Wake Forest University Health Sciences Institutional Review Board (IRB00075887). Participants provided informed consent or assent before engaging in the interview by reviewing and signing a REDCap form. This REDCap form included a brief overview of the study, information about study procedures, details regarding the risk level of the study (minimal risk), potential risks (eg, breach of confidentiality) and benefits (eg, helping others), compensation information, and ways to contact the principal investigator and Institutional Review Board. For adolescents, parents were initially contacted and asked to provide permission for the adolescent to participate. Study staff contacted adolescents directly only after parental permission was provided.

All data were collected remotely using platforms designated as compliant with the Health Insurance Portability and Accountability Act (HIPAA) and stored on secure, password-protected servers accessible only to study staff. Individual interview recordings were transcribed and deidentified for analysis. All information linking participants to their data will be deleted upon publication of the primary study outcomes.

To compensate participants for their time, a US $40 digital gift card was emailed to the participant following the interview.

### Individual Interviews

All interviews were conducted via Cisco Webex. Participants were asked to complete the interview in a quiet, private space. Only KMI and the participant were present during the interview. At the beginning of each interview, KMI provided context regarding the project goals and the work completed thus far (ie, co-design workshops with AYACS and providers). Most of the interview was dedicated to showing participants the mid-fidelity prototype of the tool, explaining how each page of the prototype might work, and requesting targeted feedback (see Multimedia Appendix 1 for example interview questions and associated slides; questions were not pilot-tested before study commencement). At the end of the interview, participants were also asked to provide any additional feedback that had not been shared during the interview.

### Data Processing and Analysis

Individual interviews were recorded and auto-transcribed using Cisco Webex software. Given these recordings, field notes were not made by KMI, and transcripts were not returned to participants for comment or correction. Transcripts were cleaned by study staff. The original audio/video recordings were consulted as needed for clarification. Our sample size of 14 is within the recommended number of interviews needed to reach saturation in qualitative research [32] and in other work focused on developing digital interventions for young cancer survivors [33].

Guided by the Rigorous and Accelerated Data Reduction technique for rapid qualitative analysis [34], we derived themes from the data. Interview transcripts were organized by interviewee, aspect of the digital tool in question, and specific interview questions in an Excel spreadsheet (Microsoft Corporation) to serve as the phase 1 data table. Next, 2 coders (KMI and GW) collectively brainstormed an overarching question to determine which responses would be included in the reduced phase 2 data table: What changes do we need to make to this prototype to more fully integrate AYA needs and preferences for a digital tool to help them manage depressive symptoms? A response was included in the coding process only if it answered this question directly. The coders independently determined whether each response met this criterion. They then compared and discussed their decisions to form a final list of responses to be included in the coding process. During this process, the coders also identified data they believed would be best captured quantitatively. This approach was most often warranted when the interviewer presented the interviewee with options from which to choose, allowing the interviewee to express a preference. For example, participants were shown several options for what the mood rating scale might look like and were asked how often they would be willing to track their mood. Choosing to capture data quantitatively did not preclude including associated context in the qualitative coding process. Quantitative data were summarized using descriptive statistics.

Next, the coders individually developed preliminary codes for the first 3 interviewees’ responses. They then compared and discussed these code ideas to determine the final codes for each response. Throughout this process, the coders attempted to use as much of the interviewees’ original language as possible to carry forward the interviewees’ sentiment. The coders then repeated this process with the next 3 interviewees’ responses, creating and adding new codes as needed until all interviewees’ responses were coded.

Following the application of codes to all interviewee responses, the coders reviewed the codes and began organizing them into themes and subthemes. Themes and subthemes were derived both empirically, based on the interview transcripts, and a priori using existing coding schemes from related work [35,36]. Coders applied themes and subthemes independently, then compared and discussed them to determine the final themes for each code, returning to the response data as needed. A final version of the data table was created to assist with the identification of major codes and illustrative quotes that helped define, refine, and illustrate the identified themes. Participants were not offered the opportunity to provide feedback on the findings.

### Design and Development of a Working Prototype

Based on feedback and recommendations from the interviews, revisions to the prototype were identified by the study team. BrightOutcome provided reprogramming of an existing digital tool (EMPOWER) [37-39] to meet the technical, functional, and aesthetic preferences of end users based on our co-design process.

Specifically, for each property of a subtheme identified through qualitative analysis, a determination was made as to (1) whether a change was needed (eg, no feature addressing a property already existed within the prototype, the existing tool being adapted, or both; the change aligned with best practices for design); (2) what change should be made (eg, the study team’s recommendation for how to best address a property through a specific adaptation); and (3) whether the change was feasible within the constraints of the budget and timeline of the current project. At times, feasibility also informed decision-making (eg, if an existing feature of EMPOWER addressed a property of the subtheme, additional features were not added). Changes that were deemed ideal by the study team but were not feasible were retained as potential future changes.

### Adaptation of Evidence-Based Interventions for Depression

In conjunction with developing the digital platform through which content would be delivered, we adapted 4 EBIs for depression (behavioral activation, cognitive restructuring, mindfulness training, and positive psychology interventions) for digital delivery to AYACS in accordance with the first 3 steps of the planned adaptation approach [40]: (1) examine the theory of change; (2) identify population differences; and (3) adapt program content in a manner that retains the core functions of the intervention (eg, for behavioral activation, increasing engagement in enjoyable activities) while allowing for tailoring (eg, using examples and stories to demonstrate how core functions such as engagement in enjoyable activities might look different for AYACS than for their noncancer peers).

## Results

### Participants

Fourteen AYACS participated in the individual interviews. At the time of the interview, AYACS ranged in age from 15 to 38 years (mean 25.7, SD 9.14 years). AYACS were diagnosed with cancer between the ages of 13 and 37 (mean 23.4 years, SD 8.9 years) and were between 1 and 55 months posttreatment (mean 23.7 months, SD 16.1 months). The majority of AYACS identified as non-Hispanic White, female, and heterosexual (see Table 1 for additional details).

**Table 1:** Demographic and clinical characteristics of adolescent and young adult cancer survivors who participated in individual interviews (N=14).

Characteristic		
	Group	Values, n (%)
**Sex**		
	Female	9 (64)
	Male	5 (36)
**Race**		
	Black/African American	2 (14)
	Native American/Alaska Native	1 (7)
	White/Caucasian	9 (64)
	Mixed racial background	1 (7)
	Other race	1 (7)
**Ethnicity**		
	Hispanic	1 (7)
	Non-Hispanic	13 (93)
**Level of education**		
	Some high school	3 (21)
	High school diploma or equivalent	2 (14)
	Some college	2 (14)
	College graduate	4 (29)
	Graduate degree	2 (14)
**Sexual orientation**		
	Heterosexual	13 (93)
	Prefer not to answer	1 (7)
**Cancer type^a^**		
	Bone	2 (14)
	Brain	1 (7)
	Breast	2 (14)
	Gastrointestinal stromal tumor	1 (7)
	Leukemia	1 (7)
	Non-Hodgkin lymphoma	3 (21)
	Sarcoma	1 (7)
	Testicular	1 (7)
	Thyroid	3 (21)
**Marital status**		
	Single, never married	8 (57)
	Married	6 (43)
**Have children**		
	Yes	4 (29)
	No	10 (71)
**Household income (US $)**		
	Less than 25,000	0 (0)
	25,000 to 34,999	2 (14)
	35,000 to 49,999	0 (0)
	50,000 to 74,999	2 (14)
	75,000 to 99,999	2 (14)
	100,000 to 124,999	2 (14)
	125,000 or more	1 (7)
	Do not know/prefer not to answer	4 (29)
**Health insurance**		
	Private	9 (64)
	Public	4 (29)
	None	2 (14)

^a^Percentages add up to >100% because 1 participant reported being diagnosed with both bone cancer and non-Hodgkin lymphoma.

### Quantitative Outcomes

#### Overview of Themes

Participant preferences captured quantitatively fell into 4 key areas: (1) mood tracking; (2) content presentation; (3) user input; and (4) duration of use.

#### Mood Tracking

To evaluate preferences for mood tracking, participants were asked what format they would prefer for the response options for mood tracking, how often they would want to track their mood, and how mood data should be displayed. The majority of participants (n=10, 71%) indicated that they preferred an option that included emojis and would be willing to track their mood at least once per day (n=11, 79%). Users preferred to be able to review a month’s worth of data (n=7, 50%) via a calendar display that showed their mood each day using an emoji (n=8, 57%).

#### Content Presentation

Regarding content presentation, participants indicated that they would want to view stories of the experiences of other AYACS as live-action videos (n=12, 86%), with a slight preference for AYACS themselves sharing their stories (n=7) rather than actors (n=5). However, for educational information, participants suggested that presenting the content in multiple formats (ie, a combination of video, audio, and text) would be preferred (n=12, 86%). There was no clear consensus on whether live-action or animated videos would be preferred for educational information.

#### User Input

More participants reported that mood and activity tracking should be conducted using multiple-choice questions (n=7, 50%) rather than free-text response boxes (n=3, 21%) or a chatbot (n=4, 29%). In this context, 5 participants also suggested including an “Other” option that allows for free-text responses. Half of the participants indicated that they would be willing to complete a practice activity at least once per day (n=7, 50%). Participants suggested that multiple-choice questions should also be used when reflecting on the stories of other AYACS (n=11, 79%), whereas free-text responses were preferred for reflecting on their own experiences (n=10, 71%).

#### Duration of Use

When asked how long participants would want to spend completing a lesson like the one presented in the example, responses ranged from less than 5 to 90 minutes, with most responses (n=8, 57%) falling between 5 and 30 minutes. When asked how long they could see themselves using a tool like this, responses ranged from 1 month to several years, with many participants suggesting that it would depend on factors such as whether they found the tool helpful, how they were doing medically and psychologically, and whether they had developed relationships with other users.

### Qualitative Outcomes

#### Overview of Themes

Participant preferences captured qualitatively fell into 4 themes: (1) features to promote user engagement; (2) tailored content presentation; (3) perceived usability; and (4) interface design.

#### Theme 1: Features to Promote User Engagement

##### Overview of Subthemes

Participants provided feedback regarding potential changes to existing features included in the prototype and suggested the addition of new features to help ensure that users remain engaged with the digital tool. Within this theme, 4 subthemes were identified: communication, options, reinforcement, and value.

##### Subtheme 1.1: Communication

Participants emphasized that the opportunity to connect with other cancer survivors around their age would increase their engagement with the tool. They suggested that AYACS would be more interested in conversations about the unique experience of being an AYACS rather than in conversations tied directly to the content presented through the tool (see the “Subtheme 4.3: Organization” section).

Interviewer: What would make cancer survivors your age want to use this tool?

Participant: Being able to interact and connect with other people who have gone through the same thing.

16-year-old female bone cancer survivor

If I was having a conversation or if there was a discussion board and everyone was really engaged in it, that might be really fun, and I might do that for a while. So, I'd maybe spend 5 minutes on it but then there's also like other stuff going on in the app that's more fun.

25-year-old female sarcoma survivor

Furthermore, participants expressed a desire for the tool to provide opportunities to connect with providers. They suggested that knowing a provider would review their responses would increase the likelihood that they would complete activities within the tool.

If I was using this tool for this reason, I would only spend time completing it and giving it attention if I knew that someone on the other side was going to take it and use it somehow. If it's going to help form a conversation with a provider next week. But if I'm just doing it because someone who created an app think it's helpful, I'm probably not going to put effort into it. ...But if you were my provider and I knew we had a quarterly meeting, then I would type something in because then I would anticipate that they would view it prior to our meeting.

34-year-old male non-Hodgkin lymphoma survivor

However, privacy also emerged as an important consideration; some participants felt it would be helpful to reflect on and process their experiences in a space that could not be viewed by others.

I don't want everyone to see what I'm doing. I know we're going through similar situations, but sometimes I like to keep stuff to myself.

20-year-old female leukemia survivor

##### Subtheme 1.2: Options

Participants thought that users should be allowed to view content in whatever order they choose. Having the option to access content as needed would allow them to tailor what they access to their needs in the moment.

Especially for me, with my cancer that I went through, I didn't really feel the depression at first. I felt like I needed more support so if you're not feeling like a certain way and that's the beginning, I think it wouldn't explain a lot of it until you get into it. So, I think if you could click at the point where you're at, it'll help you a bit more.

32-year-old female thyroid cancer survivor

Some participants were concerned that having the option to view any of the content they would like might be overwhelming, and recognized that there are individual differences in preferences for the level of autonomy.

I always appreciate freedom, but I know that most of my students, they get panicked when they have freedom. So, my preference is certainly not a universal preference.

35-year-old male non-Hodgkin lymphoma survivor

Participants also suggested that any practice activities in the tool should be optional.

I would like to be able to go try it and wait before I come back to it. Because I want to be able to implement those skills. But you might have people wanting to go straight through. So maybe give me the option. Maybe have a pop up notification that says you can choose to try it that week or you can go straight through.

20-year-old female leukemia survivor

##### Subtheme 1.3: Reinforcement

The most common recommendation from participants regarding how to ensure engagement with the tool was the use of gamification. Most AYACS felt that gamification could make the tool more interesting and fun, whereas others felt it might be inappropriate given the subject matter of the tool.

If it were to be something where you could accumulate points or something like that, you could redeem those points later on. And it would make it more interesting and more fun for them to answer those questions and put a little bit more effort into it.

32-year-old female thyroid cancer survivor

In addition to gamification broadly, participants suggested various reinforcement features that they had found helpful in other tools to ensure engagement, including the use of streaks, reminders, and a progress bar.

A lot of other apps use this, but you could use a streak, like how many days you've used it in a row, it could be on the calendar. That motivates people to keep doing it cause they don't want to lose their streak.

15-year-old male gastrointestinal stromal tumor survivor

I could just set it to where it goes off on my phone. I just click on it and choose how I'm feeling at that moment. So having a reminder notification to do it would be helpful.

25-year-old female sarcoma survivor

Interviewer: What would make it more likely that you would complete a session like this?

Participant: A progress bar that shows how far you are within all the modules.

16-year-old female bone cancer survivor

However, participants also emphasized that it would be important to be able to turn off or manage the frequency of these engagement-promoting features because a variety of factors outside the tool may affect their use of it (eg, cancer trajectory, severity of disease, daily activities, and personal preferences).

I don't know if it's possible for the user to set their own settings or to mute it for the day or something like that. So they're not randomly being prompted to check in on the days that they're not doing well. That might be a good option. [later, in response to another question] I'm in a weird spot right now. I've had active treatment for 3 years so that was a very big part of my life. And now I don't have active treatment and there are days when I'm doing quite well and then there are days when things surface and I can make sense of them and then there are days when things surface and I just can't make sense of it. I would like to not have the constant reminder. When I'm doing well, just let me do well. And when things are difficult, it would be nice to have a resource.

35-year-old male non-Hodgkin lymphoma survivor

##### Subtheme 1.4: Value

Some participants struggled to see the value of the tool for themselves and suggested that providing education about why they might find the tool useful early on would increase the likelihood that they would engage with it.

Not to diminish the tool, I just don't see the value for me personally. That's why I wouldn't spend a lot of time on it. Maybe if I was educated on the value it can provide for someone like you, maybe. But I don't think getting tokens or stars is going to change my mind.

34-year-old male non-Hodgkin lymphoma survivor

Additionally, participants noted that if they found the tool helpful, they would be more likely to use it in the long term.

If I feel like the app is working, I'm more likely to use it long term. If I feel like it's not doing anything, I'm more likely to delete it and just go on without it.

20-year-old female leukemia survivor

#### Theme 2: Tailored Content Presentation

##### Overview of Subthemes

Participants provided feedback regarding how to best tailor content to this target population. Within this theme, 7 subthemes were identified: accessibility of activities, authenticity, avoiding discouragement, duration, maturity level, normalization, and use of multimedia.

##### Subtheme 2.1: Accessibility of Activities

Participants expressed concern that certain activities, such as a walking meditation, might be difficult for some AYACS to complete. They suggested including modifications for these activities if they are incorporated into the final tool.

With mindful movement, there could be modifications in case someone can't do what they're doing in the video.

15-year-old male gastrointestinal stromal tumor survivor

##### Subtheme 2.2: Authenticity

Participants emphasized the importance of authenticity in the portrayal of information, as well as in the portrayal of AYACS in images throughout the tool. They also noted the importance of experts truly being experts rather than actors performing a role.

Hearing it come from an actual person would feel more understanding and it feels more human.

16-year-old male brain cancer survivor

Similarly, they desired imagery that conveyed the authentic, messy experience of having cancer as a young person rather than “branded cancer.”

A lot of health care and cancer imagery is very much not like the experience. Even though I had people on my side, at no point was there someone just casually over my shoulder, holding my hand, and we were wistfully looking off into the distances with two cups of coffee...I just feel like my cancer experience was way more messy than this. So removing the image would be helpful here. If you actually do real interviews with real people and have a real photograph, then maybe that gets past it. But this is branded cancer - I bet that person doesn't have cancer. They just have a nice scarf on their head.

35-year-old male non-Hodgkin lymphoma survivor

##### Subtheme 2.3: Avoiding Discouragement

Throughout the tool, AYACS emphasized that users should not find the content discouraging. This concern was evident in their considerations of how mood data should be displayed over time, as well as how incorrect answers to multiple-choice questions should be addressed.

If you're going through a slump and it [the line graph] just keeps going lower and lower, that can be discouraging.

21-year-old female non-Hodgkin lymphoma survivor

I think it's okay cause they can go back and reflect on it and figure out what actually was the issue. Maybe after 2 wrong guesses, just kind of give them the correct answers so they're not just sitting there. They might get frustrated after a couple times. Younger people might not get what is actually starting the event of them getting depressed and it helps them reflect on it but then also not getting frustrated after 1 or 2 guesses.

36-year-old female breast cancer survivor

##### Subtheme 2.4: Duration

AYACS indicated that it would be preferable to have variation in lesson duration. They suggested that this would make the tool feel less “sterile” and allow them to choose a lesson based on its duration.

I would probably appreciate them being different lengths so that I can pick based on the amount of time that I'm either emotionally or physically able to invest.

35-year-old male non-Hodgkin lymphoma survivor

AYACS also noted that the duration of practice activities would affect the frequency with which they were willing to complete them and suggested that longer activities should be required less frequently than shorter activities.

If you ask them to do it every day, I'd keep it shorter, like 5 minutes. Or if they're longer, you could do 3 times a week or twice a week.

15-year-old male gastrointestinal stromal tumor survivor

Finally, there was no clear consensus on how long users might want to engage with the tool. While some felt that it might be most helpful years after the completion of treatment, when less support is available, others felt that the duration of their use would depend on their health, implying that it might be more helpful if their cancer recurred.

I think it would need to be available to people for a long time. In my case, it was a couple of years until I realized the trauma I'd been through and needed help getting through it. And at that point, I wasn't even seeing a doctor anymore. I didn't have any support.

37-year-old male testicular cancer survivor

Interviewer: How long can you see yourself using a tool like this?

Participant: I think it would depend on how I'm doing medically.

25-year-old female sarcoma survivor

##### Subtheme 2.5: Maturity Level

AYACS recognized that preferences for content delivery may vary across the wide age range targeted by the tool. However, participants most consistently cautioned against presenting the content in a manner that would be perceived as juvenile.

A lot of the people you'll be dealing with are going to be very emotional because of what they're dealing with or have dealt with, but they've also matured in ways that their peers haven't. So, for me personally, I would disengage if it was too juvenile in nature.

35-year-old male non-Hodgkin lymphoma survivor

##### Subtheme 2.6: Normalization

It was important to AYACS that the tool normalize their experience, particularly regarding the impact of cancer on one’s mental health.

Sometimes you may worry you're not normal during this time. So maybe listening to a psychologist talk about it, cause a lot of people don't like to ask for help.

36-year-old female breast cancer survivor

If there was a multiple choice and then you click on the choice. “Explain an event” - is it family, school, friends, social, financial, whatever. Then let's say you click “financial” and then it pops up a bubble that affirms that's a normal thought to have, or studies show that coming out, you may feel less motivated in your career. That makes sense to me. Even if it's a robot, at least someone is listening.... Just a blurb that affirms that you're not crazy.

34-year-old male non-Hodgkin lymphoma survivor

##### Subtheme 2.7: Use of Multimedia

AYACS suggested that using animation throughout the tool would make it more engaging, interactive, and fun. Additionally, AYACS felt that using animation to convey information about mental health could reduce stigma around mental health concerns and make the information easier to understand.

I like animated stuff. I think that's more engaging than a psychologist going through something.

15-year-old male gastrointestinal stromal tumor survivor

You go through different employer trainings. When something is animated, it takes the taboo or the stigma off of certain topics. It makes it more easy to understand. As opposed to reading it or having a formal psychologist reading it.

34-year-old male non-Hodgkin lymphoma survivor

While each participant expressed preferences for content presentation, they often suggested presenting content in multiple formats because of individual differences in how people prefer to consume information.

I know for myself, I like to read things. I don't like when someone else is reading to me, but everybody's different.

20-year-old female thyroid cancer survivor

#### Theme 3: Perceived Usability

##### Overview of Subthemes

Participants suggested strategies to enhance the perceived usability of the tool. Within this theme, 4 subthemes were identified: accessibility, clarity, navigation, and user input.

##### Subtheme 3.1: Accessibility

Participants recognized the importance of ensuring that content would be accessible to all users. In particular, they emphasized that it would be helpful to present information in multiple formats (ie, text and video or audio) and that text should be easy to read.

Having the transcript is good. You never know when you're going to be somewhere where you can't hear or watch, and it's good for accessibility too.

37-year-old male testicular cancer survivor

I think that the white color of the words could be hard to see. Because it's so light in the green.

25-year-old female sarcoma survivor

##### Subtheme 3.2: Clarity

Participants expressed a desire for greater clarity from the tool in several areas, most notably the name of the tool, the module overview, and how the tool would work. Specifically, participants expressed concerns about the use of the acronym “AYA” in the name of the tool.

iManageAYA would definitely make me curious, but maybe it should be explained more, like what AYA is.

20-year-old female thyroid cancer survivor

With regard to the module overview page, participants suggested using names that were more clearly distinct from one another and adding a description for each module. This would help users better understand which module they may want to engage with.

The Doing & Feeling, Thinking & Feeling, and Being & Feeling, maybe I'm not very educated in this space, but I don't understand how those are very different. Why would I click on one versus the other?...I can't distinguish between those three.

34-year-old male non-Hodgkin lymphoma survivor

I think it would be helpful to have descriptions of what they are rather than just those little snippets of Doing & Feeling and Being & Feeling. Especially if it was somebody who was a lot younger, because they might not have heard of those things before....it should be something a little bit more so that you know exactly what you're clicking on or even if you do keep the titles, put something under it in parentheses that kind of sums up what that particular section is.

38-year-old female breast cancer survivor

Finally, participants noted that it would be helpful to include more information about how the tool’s features work to prevent confusion.

Maybe you could have a note that says that once you complete a session, it will unlock the next one. That way people don't think that they can't get into it and that there's something wrong with the app.

32-year-old female thyroid cancer survivor

##### Subtheme 3.3: Navigation

Participants indicated that it would be important for users to be able to leave the tool and easily return to where they left off. Suggested strategies to achieve this included ensuring an easy log-in process and adding a bookmark feature.

Because I have a million passwords and it's hard to keep up with all of them. So having an easy log in process is key.

32-year-old female thyroid cancer survivor

I would definitely use [a bookmark feature] because I'm not as consistent as I'd like to be. So I can look at it for like 2 or 3 days consistently, and then I get busy and then I would like to remember where I can come back to.

2-year-old female leukemia survivor

Participants also requested enhancements to the daily check-in question, such that additional questions would be asked of the user. Participants suggested that these questions could be added to all check-in questions as an optional addition, asked only if a pattern of negative emotions is detected, or asked at predetermined intervals.

Another option would be...after you click one [an emoji], you can write a bit about exactly how you're feeling. But the writing would be optional.

16-year-old male brain cancer survivor

But if somebody is interested and struggling through the emotions post-treatment and it's just a quick notification, you could just click it once a day or something like that. If the trend starts, or if somebody consistently does a certain emotion, then maybe it can dig deeper after a week or two and maybe a deeper dive monthly.

34-year-old male non-Hodgkin lymphoma survivor

Finally, participants suggested making modifications to the bottom navigation menu of the tool, including removing the text and reorganizing it depending on where users are in the tool.

Something about the dashboard, ldlearn, discuss, connect, something just looks a little off. Maybe if you just use the logos there, cause you can infer from the logos what it'd be.

15-year-old male gastrointestinal stromal tumor survivor

I think once you click on a Learn module, the anchor (Dashboard, Learn, Discuss, Connect) could all be reorganized to be specific to what is being engaged with in that Learn module. And then you could keep the Dashboard. But if I'm in a Learn module, I don't really need to see my Inbox.

35-year-old male non-Hodgkin lymphoma survivor

##### Subtheme 3.5: User Input

Overall, participants reported preferring simplicity over complexity for user input, particularly when the goal is for users to engage in reflection about the content rather than to collect back-end data. Yet, participants recognized that there are benefits and limitations to both free-text and multiple-choice responses.

If it's just about the person checking in with themselves, then just keep it simple. Whereas, if you're actually trying to collect data, then you need to get into the nuance. Like you can be angry and happy at the same time.

35-year-old male non-Hodgkin lymphoma survivor

I like the multiple choice, but then I realize that it would be hard to put all the different emotions that you would have in...I feel like it would be harder to sit there and write out everything and people may not have the time. Versus if they could just choose something, they're more likely to do it cause it's quicker and faster. So I like a way that they can quickly choose something, but like I said, I don't know how you would do that cause that would be hard with so many different emotions. But I do feel like if you did a lot of text, people would not engage in it just because of the time that it would take to sit there and write all that out.

36-year-old female breast cancer survivor

Participants emphasized that it would be useful to have multiple options for inputting responses to free-text questions, as this would allow for more accurate expression of an individual’s experience.

I also like the different words because sometimes you struggle to find a certain word for pain or how you're feeling and sometimes you're empty, like you don't have an emotion for some reason and so it's more than just feeling terrible. You just kind of feel numb...I know it's a lot, but when you have a lot of options to express yourself, you're more likely to convey how you're actually feeling...Maybe even having the option to put a picture there so you can look back on it and remember how you were feeling.

25-year-old female sarcoma survivor

I've been playing with the idea of how to get students to engage with their personal thought life. I have found that there is resistance to typing with your thumbs. But, as soon as I remind them that most phones now have voice activation auto-text, I get a lot more written responses simply because they remember that they can push the microphone and it will type for them. So maybe having a reminder like that would be good. People will think differently and record their thoughts differently. So since this is for them, I would give them options. Maybe they hand write it out on paper and take a photograph and upload it. Or record an audio clip. But I think those sorts of accommodations, if it's possible to achieve, I think it would make for a more personal experience than, you know, type a minimum of 150 words.

35-year-old male non-Hodgkin lymphoma survivor

#### Theme 4: Interface Design

##### Overview of Subthemes

Participants provided suggestions on how to improve the design of the user interface. Within this theme, 4 subthemes were identified: coherent theme, color scheme, organization, and text.

##### Subtheme 4.1: Coherent Theme

Participants indicated that they would like the tool to have a consistent look and feel, with continuity across images.

Aesthetically, the continuity isn't there between each image. It looks like different artists are making each of them. I personally don't care about branding, but it does make it a little bit disjointed. It's like, in one you're at a Trader Joe's and another you're at Kroger. I would tidy those up. The first one has hardly any facial features and then the last one, Thinking & Feeling, it's emphasizing facial features, but in a very cartoon-y way.

35-year-old male non-Hodgkin lymphoma survivor

##### Subtheme 4.2: Color Scheme

Participants suggested incorporating brighter colors into the design of the tool and allowing users to customize the color scheme according to their preferences.

They're more neutral colors. It wasn't anything that really popped out. So maybe I would incorporate bright colors as well.

38-year-old female breast cancer survivor

I think it would be nice to be able to customize the theme or the colors, so it's the person's favorite color.

21-year-old female non-Hodgkin lymphoma survivor

##### Subtheme 4.3: Organization

Participants indicated that they would prefer discussion boards to be organized by survivor groups (eg, specific cancer diagnosis, treatment type) rather than having a discussion board for each content area.

People would always have questions, so I don't know that you're going to have a problem with people interacting. Cause people always worry about their health, especially afterwards. I feel like I worry all the time and I don't know if it's normal. So I would get on there and read to see if I'm the only one going through this. I feel like a discussion board where they can talk about anything rather than ones specific to the content would be more useful to people...Maybe the different types of cancers so that you can have somebody that's going through what you have, some of the same treatments and stuff like that. I would rather connect with somebody that went through breast cancer just like I did rather than somebody with a different kind of cancer where we went through two different treatments.

36-year-old female breast cancer survivor

##### Subtheme 4.4: Text

Participants requested that information presented in text be visually appealing.

I think it's [the transcript] very bland. I would add a different font because I feel like I'm just reading a paragraph of text. Maybe add some colors too.

20-year-old female thyroid cancer survivor

### Design and Development of a Working Prototype

**Figure 1:**
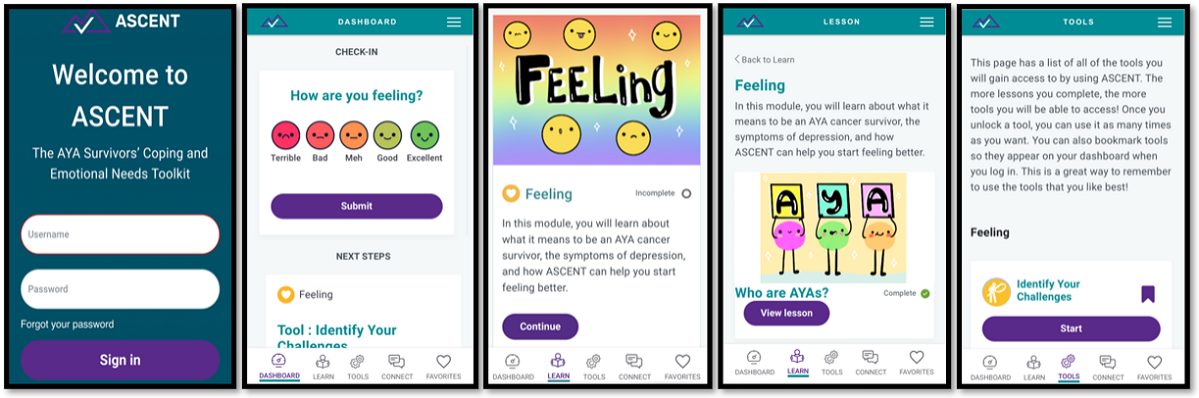
Screenshots of the ASCENT (AYA Survivors’ Coping and Emotional Needs Toolkit) working prototype.

Based on insights obtained during individual interviews, a working prototype was developed by reprogramming an existing digital tool (EMPOWER) [37-39] by BrightOutcome, a health care technology company. While much of the working prototype aligned with the design of the mid-fidelity prototype and EMPOWER, modifications were informed by quantitative findings and themes identified in the individual interviews. To promote user engagement, we decided to provide AYACS with daily SMS text message reminders that use an encouraging tone (sent only if the user does not engage with the tool by early evening) and to provide graphical feedback on fluctuations in their mood. Regarding tailored content presentation, AYACS emphasized that content should be authentic and age-appropriate. As such, we partnered with 2 nonprofit organizations (Elephants and Tea and Cancer Rebellion) to integrate real stories from AYACS into the tool in both blog and video formats. Further, we worked with a professional animator to create age-appropriate animated psychoeducational videos. To enhance the perceived usability and accessibility of the tool, all videos include transcripts or closed captioning. We also modified the names of modules and added descriptions to provide users with a clearer understanding of what each module entails. Finally, we worked with a professional designer to improve the design of the user interface by implementing a consistent and coherent theme and using brighter colors throughout the tool. We also included artwork throughout the tool created by a professional artist who is an AYACS. Screenshots of the working prototype, now called ASCENT (which stands for the AYA Survivors’ Coping and Emotional Needs Toolkit), are presented in Figure 1.

### Adaptation of Evidence-Based Interventions

In addition to reprogramming the EMPOWER platform based on this feedback, we also adapted 4 EBIs for digital delivery. Consistent with our theoretically and empirically derived conceptual model [30], EBIs were adapted for delivery via 5 modules. Core elements of 4 EBIs (behavioral activation, cognitive restructuring, mindfulness training, and positive psychology interventions) were identified by reviewing established clinical treatment manuals, existing digital tools, and prior empirical findings. Population differences requiring adaptation were identified as the experience of having cancer, age or maturity level, and delivery via a self-guided digital platform. Intervention content was adapted to address these differences while preserving core elements and ensuring that there was no overlap between modules. The Feeling module includes 6 sequential lessons that provide general psychoeducational content about AYACS and depression, resources for identifying and managing a mental health crisis, and an introduction to how the tool’s discussion boards can be used for peer support. Each adapted EBI was included as a separate module of 6 sequential lessons based on established treatment protocols: Doing (behavioral activation), Thinking (cognitive restructuring), Being (mindfulness training), and Positivity (positive psychology interventions). Within lessons, text-based psychoeducational content was adapted into scripts for brief, educational animated videos. Interactive exercises such as Socratic questioning and goal-setting were modified for digital delivery. The content was tailored to AYACS by incorporating stories from other AYACS, using age-appropriate language and design, and incorporating features to promote engagement with the tool that were informed by user-centered design with AYACS. Table 2 provides a more detailed description of current and potential future changes to the tool based on individual interview feedback and the planned adaptation process.

**Table 2:** Current and potential future changes to the digital depression self-management informed by themes identified through qualitative analysis of individual interviews with AYACS^a^.

Theme, subtheme, and property				Working prototype		Potential future changes		
**Theme 1: Features to promote user engagement**								
	**Communication**							
		Opportunities to interact with other AYACS using the tool would increase engagement.	A discussion board will be included for each content area of the tool^b,c^.		N/A^d^			
		The tool should allow patients to connect with a mental health provider.	Information about crisis management will be provided as part of the psychoeducational module^e^.		Add the ability to connect with local providers.			
		While users wanted opportunities to engage with others through the tool, they also wanted some aspects of it to be private.	Users will be provided with the option to share their responses to open-ended questions to the relevant discussion board^c^.		N/A			
	**Options**							
		Participants felt that users should be able to choose in what order they view content unless it needs to be presented sequentially.	Users will be able to complete modules in any order at their own pace; however, within modules, content will be presented sequentially^b^.		N/A			
		Participants expressed concerns that receiving all content at once may be overwhelming.	Users will only have access to 1 new lesson per module at a time^b^.		N/A			
		Allowing users to choose the order in which they view content allows them to tailor it to what they need in the moment.	Users will be able to complete modules in any order at their own pace^b^.		N/A			
		Completion of questions, practice activities, etc throughout the tool should be optional.	N/A		Consider making only certain practice activities that are essential for the benefit required; this will require additional empirical information.			
	**Reinforcement**							
		Gamification could make the tool more interesting and fun, but it may be inappropriate given the nature of the content.	Gamification tactics shown to be efficacious in and appropriate for health behavior change interventions will be used (eg, motivational messages)^e^.		N/A			
		Participants suggested including various features to increase user engagement: motivational messages, streaks, rewards, progress bar, and reminders.	Daily reminders to use the tool will be sent using a motivational tone^e^.		Consider adding features such as streaks, progress bars, and rewards.			
		A variety of factors impact the frequency at which users want reminders to track their mood and activities. These include cancer trajectory, severity of disease, daily activities, and personal preference.	Reminders will be provided once daily^e^.		Additional tailoring/personalization of reminder frequency.			
		Users should have the option to turn features meant to promote engagement on or off.	N/A		Consider providing an option for users to turn reminders off; this will require additional empirical information.			
	**Value**							
		Education on the value of the tool will increase AYACS motivation to engage with it.	N/A		Provide education on the value of the tool once supported by empirical data.			
		Experiencing improvements in symptoms will increase the duration of use.	Users will receive graphical feedback on how their mood has fluctuated each time they track it in the tool^c^.		N/A			
**Theme 2: Tailored Content Presentation**								
	**Accessibility of activities**							
		Certain activities may be difficult for some survivors to complete (eg, walking meditation); modifications should be provided if these activities are included.	A curated group of activities was chosen to ensure individuals of all physical abilities would be able to complete them^e^.		N/A			
	**Authenticity**							
		Experts and AYACS should be portrayed in an authentic manner.	Information about the expertise of the team involved in the development of the tool will be provided^c^. Real stories from AYACS will be integrated to illustrate concepts^e^.		N/A			
	**Avoiding discouragement**							
		Information should not be presented in a manner that could be considered discouraging or demotivating.	An encouraging and supportive tone will be used throughout the tool and in all reminders^e^.		N/A			
	**Duration**							
		Lesson duration should be variable.	While all lessons include similar parts, the length of time to complete each part will be variable^e^.		N/A			
		The duration of practice activities should influence the frequency of required completion.	Longer practice activities will require fewer repetitions before unlocking additional content^e^.		N/A			
		Preferred duration and timing of use are variable.	Users will be asked to engage with the tool for 6 weeks, as each module has 6 lessons^b^.		Modify the duration of the study if needed to ensure users can complete all content.			
	**Maturity level**							
		Content should not be presented in a manner that could be considered juvenile.	A professional designer and an artist will assist with ensuring visuals are age-appropriate^e^.		N/A			
	**Normalization**							
		The tool should provide normalization regarding the impact of cancer on AYACS mental health.	A lesson focused on the relationship between cancer and depressive symptoms in AYACS will be included in the psycho-educational module^b^.		N/A			
	**Use of multimedia**							
		Using animations will make the tool more engaging, interactive, and fun, as well as reducing stigma and enhancing comprehension.	Psychoeducational information will be provided via animated videos^e^.		N/A			
		Content should be presented in multiple formats to account for individual differences.	All video content will include transcripts and closed captions^e^.		N/A			
**Theme 3: Perceived Usability**								
	**Accessibility**							
		Information should be presented in multiple formats.	All video content will include transcripts and closed captions^e^.		N/A			
		Text should be easy to read.	A professional designer will ensure the tool uses fonts and colors that are easy to read and visually appealing^e^.		N/A			
	**Clarity**							
		Potential users may not know what AYA^f^ in the name iManage AYA stands for.	The tool was re-named ASCENT^g^, which stands for the AYA survivors coping and emotional needs toolkit; the meaning of AYA will be described in the first lesson of the psychoeducational module^e^.		N/A			
		Rename modules to allow for clearer differentiation between topics; include brief descriptions of each module.	Modules were renamed to provide additional clarity^e^. A brief description was added for each module^e^.		N/A			
		Include more information about how the practice activities work to prevent confusion regarding lack of access.	As part of research studies, users will be required to complete an orientation call in which they learn how to use the tool^e^.		Provide potential users with a manual or introductory video, or both, that describes how to use the tool.			
	**Navigation**							
		Users should be able to leave the tool and easily come back to where they left off.	Users will be able to ask the tool to remember their log-in information^c^. Features such as bookmarks and favorites will support ease of navigation within the tool^e^.		N/A			
		Include additional questions in the daily check-in.	N/A		Add questions to the daily check-in at either predetermined intervals or based on consistent low mood.			
		Remove text from the navigational menu.	N/A (not in alignment with best practices for web design).		N/A			
		Reorganize the navigational menu depending on where the user is in the tool.	N/A (not in alignment with best practices for web design).		N/A			
	**User input**							
		User input should be as simple as possible, particularly when the goal is for users to engage in reflection on content.	Multiple choice questions will be prioritized as much as is feasible to ensure ease of input^e^.		N/A			
		Users should have multiple options for inputting responses to free-text questions.	The web-based application is compatible with native talk-to-text features^c^.		Add photo upload, use of GIFs, etc as response options for open-ended questions.			
**Theme 4: Interface Design**								
	**Coherent theme**							
		The tool should have a consistent look and feel with continuity between images.	A consistent icon set, color scheme, and artistic style will be used throughout the tool^e^.		N/A			
	**Color scheme**							
		Incorporate brighter colors into the design.	Brighter colors will be incorporated into the design of the working prototype^e^.		N/A			
		Allow users to customize the color scheme of the tool.	N/A		Allow users to customize the color scheme of the tool.			
	**Organization**							
		The discussion board should be organized by disease or treatment characteristics rather than content area.	N/A (not appropriate for planned full factorial trial in which it is necessary to prevent cross-contamination between conditions).		Reorganize discussion boards in the optimized version of the tool.			
	**Text**							
		Text should be visually appealing.	A professional designer will ensure the tool uses fonts and colors that are easy to read and visually appealing^c^.		N/A			

^a^AYACS: adolescent and young adult cancer survivors.

^b^Existing feature of the mid-fidelity prototype.

^c^Existing feature of EMPOWER.

^d^N/A: not applicable.

^e^Change based on interview feedback.

^f^AYA: adolescent and young adult.

^g^ASCENT: AYA Survivors’ Coping and Emotional Needs Toolkit.

## Discussion

### Principal Findings

This study aimed to explore the preferences of AYACS for the design and content of a digital self-management tool for depressive symptoms. The findings indicate that AYACS highly value personalization, flexibility, and peer support in digital interventions. Specifically, participants emphasized the importance of tailored content that resonates with AYACS’ lived experiences of cancer survivorship, opportunities for interaction with peers facing similar challenges, and features that allow flexible engagement with the tool. These preferences underscore the need for a tool that is uniquely tailored to the needs of AYACS.

### Comparison With Prior Work

This study provided key insights into the unique needs and preferences of AYACS for digital mental health interventions. First, the findings will inform the development of a working prototype of the digital tool being developed by our team and have broader implications for researchers seeking to develop interventions tailored to this population.

A second strength of this study is the use of user-centered design methods with AYACS. User-centered design has been increasingly recognized as an important strategy for informing the development of digital mental health interventions in the general population [41]. Further, user-centered design methods have been successfully used with young people with cancer to inform the development of health care transition and symptom self-management interventions [42-44]. However, such methods have not explicitly been used to develop digital mental health interventions for AYACS [37,45,46], although AYACS have provided input that has guided the development of these tools. By relying on established methods from the field of human-centered design to guide the inclusion of AYACS in informing the design of our tool, we have gained valuable insights into AYACS’ needs, motivations, and preferences for digital mental health tools. Ultimately, we believe that integrating these insights into our tool will help ensure that AYACS engage with it.

The findings of this study also underscore several important considerations for the development of digital tools for AYACS. Tailoring emerged as a key theme, with participants expressing a strong preference for content that reflects their individual cancer experiences. This aligns with findings from our prior work [30], as well as those from other groups that have identified the importance of relevant, cancer-specific content and peer stories in engaging cancer survivors in digital interventions [47,48]. AYACS’ strong preference for tailored content underscores the necessity of providing not just general mental health advice but contextually relevant information that resonates with the unique challenges of this population.

Another important finding is the need for flexibility in the tool’s design. AYACS expressed a preference for content that can be accessed at their own pace, with variation in the duration and frequency of interaction. While prior work has indicated that AYACS broadly prefer on-demand interventions [16], our findings suggest a broader desire for flexibility. Specifically, AYACS emphasized that it is important for users to be able to access the content most relevant to them at the moment when they are most ready to engage. One question this raises is how best to help AYACS make decisions about what content to access and when. Future research on digital tools should consider using adaptive trial designs such as the sequential multiple assignment randomized trial [27] to determine which interventions may be most effective for which AYACS and at what time, ultimately informing the development of a just-in-time adaptive intervention [49].

The study also highlighted the significance of peer support. Participants emphasized that having the opportunity to connect with others who have experienced similar challenges would be an important feature of the digital tool. This is consistent with prior research suggesting that AYACS place a high value on support from peers who have had cancer. Prior research indicates that peer interactions can reduce feelings of isolation and provide valuable emotional support for cancer survivors [47]. Incorporating social features, such as forums or shared experiences from other survivors, could enhance AYACS’ sense of community and support.

Finally, participants raised important ethical concerns regarding privacy when using the digital tool. In accordance with best practices for digital mental health tools, the working prototype of ASCENT was developed on a HIPAA-compliant platform with several technical safeguards in place to protect participant data. Further, participants are never required to share their personal information or responses with other users on the platform; rather, this is an optional part of ASCENT. While participants did not explicitly raise other concerns regarding the potential risks of ASCENT, this remains an important consideration in the development of such tools. Prior research suggests that while digital mental health interventions are generally considered relatively safe, the occurrence, frequency, and methods for safety data collection vary widely, making it difficult to validate this claim [50]. A recent qualitative study provided recommendations for improving the safety of interventions such as ASCENT, including acknowledging the lack of personalization, providing genuine crisis support, and anticipating and supporting deterioration [51]. Such recommendations will be critical in informing future evaluations of the effectiveness and safety of ASCENT.

### Limitations

While this study provides several important insights, it is not without limitations. Despite efforts to recruit a diverse group of participants by identifying individuals from varied backgrounds during our review of medical records, our sample does not fully reflect the broader population of AYACS in terms of race, ethnicity, gender, and sexual orientation. Future research aimed at tailoring digital health interventions for this population should seek to recruit diverse samples of AYACS via targeted recruitment strategies to ensure the generalizability of findings across diverse AYACS. However, it is worth noting that the sample was broadly representative of the typical age range and common cancer types found among AYACS. Further, while our sample size of 14 was sufficient for the planned qualitative approach, future studies are needed to test the generalizability of these findings in a larger sample of AYACS. This could occur through a stand-alone study (eg, a questionnaire designed to validate AYACS’ preferences for digital self-management tools) or as an adjunct to future trials of such tools. The qualitative methodology of this study could also have been more rigorous. For example, it is best practice to pilot-test interview guides before beginning recruitment. Further, it would have been ideal to obtain participant feedback on transcripts and identified themes and subthemes before finalizing our insights and design decisions, as well as to conduct follow-up focus groups.

### Conclusions

This study highlights the key features that AYACS value in digital self-management tools: personalization, flexibility, and peer support. These preferences underscore the need for digital interventions that are not only tailored to individual cancer experiences but also flexible in design to meet the evolving needs of AYACS over time. The broader implication of these findings is that digital health tools, when designed with these elements in mind, have the potential to improve mental health outcomes and AYACS’ overall quality of life. By offering a more personalized, flexible, and socially connected experience, digital interventions can play a crucial role in supporting this often-overlooked group of cancer survivors. Future research should continue to evaluate the impact of integrating AYACS’ preferences into digital tools on promoting sustained engagement with digital interventions, which may ultimately lead to improved health outcomes. For example, in our pilot feasibility study of procedures for a full factorial trial of ASCENT, we will collect data regarding usability, engagement, actual usage, and safety (ie, occurrence of adverse events, deterioration). Further, participants will be asked to provide feedback on the working prototype via exit interviews [52].

## Data Availability

An all-inclusive table of raw data from the transcripts is available online [53].

## Appendix

Multimedia Appendix 1 Slides presented to participants during individual interviews.

## Abbreviations

AYA: adolescent and young adult
AYACS: adolescent and young adult cancer survivors
CBT: cognitive behavioral therapy
EBI: evidence-based intervention
HIPAA: Health Insurance Portability and Accountability Act
REDCap: Research Electronic Data Capture; Vanderbilt University
