# Supplementary material for: Co-Design of a Depression Self-Management Tool for Adolescent and Young Adult Cancer Survivors: Rapid Qualitative Analysis of Interview Feedback on a Prototype
Source: JMIR Form Res. 2026 Apr 13;10:e77994. doi: 10.2196/77994 (PMC13122137; doi:10.2196/77994)
Supplement: Multimedia Appendix 1 [file formative_v10i1e77994_app1.pptx]

## Slide 1
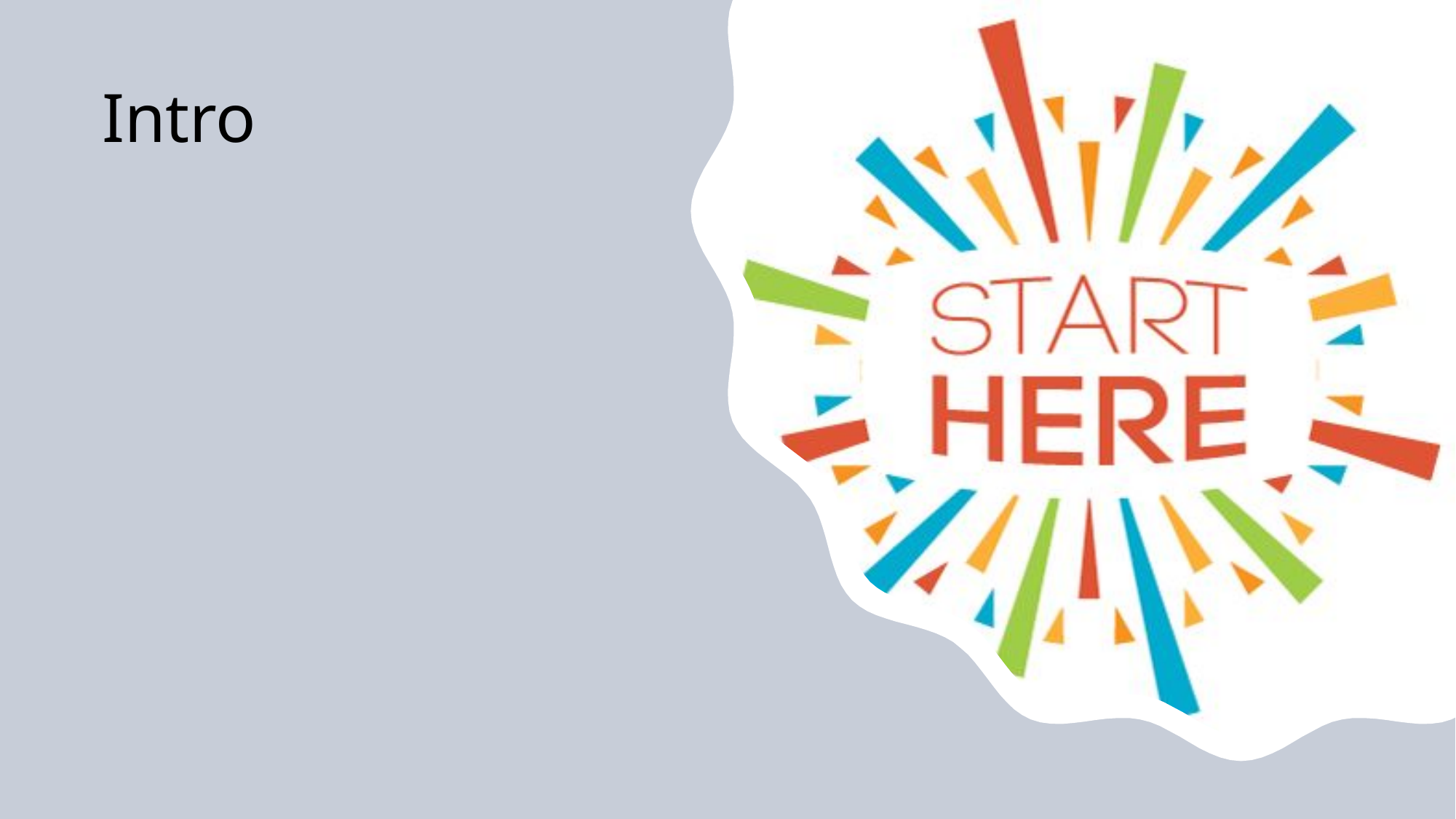

# Intro

## Slide 2
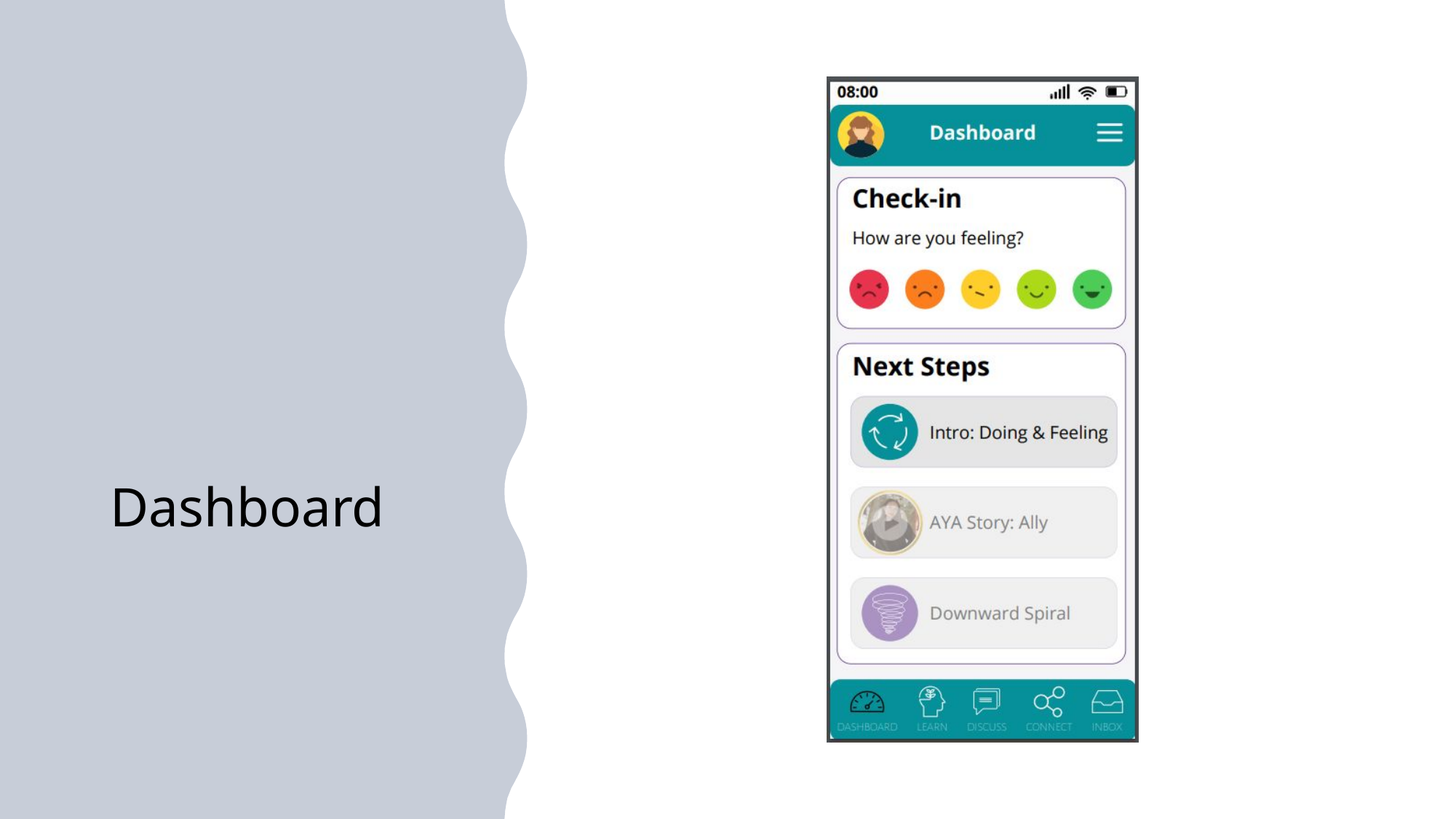

# Dashboard

## Slide 3
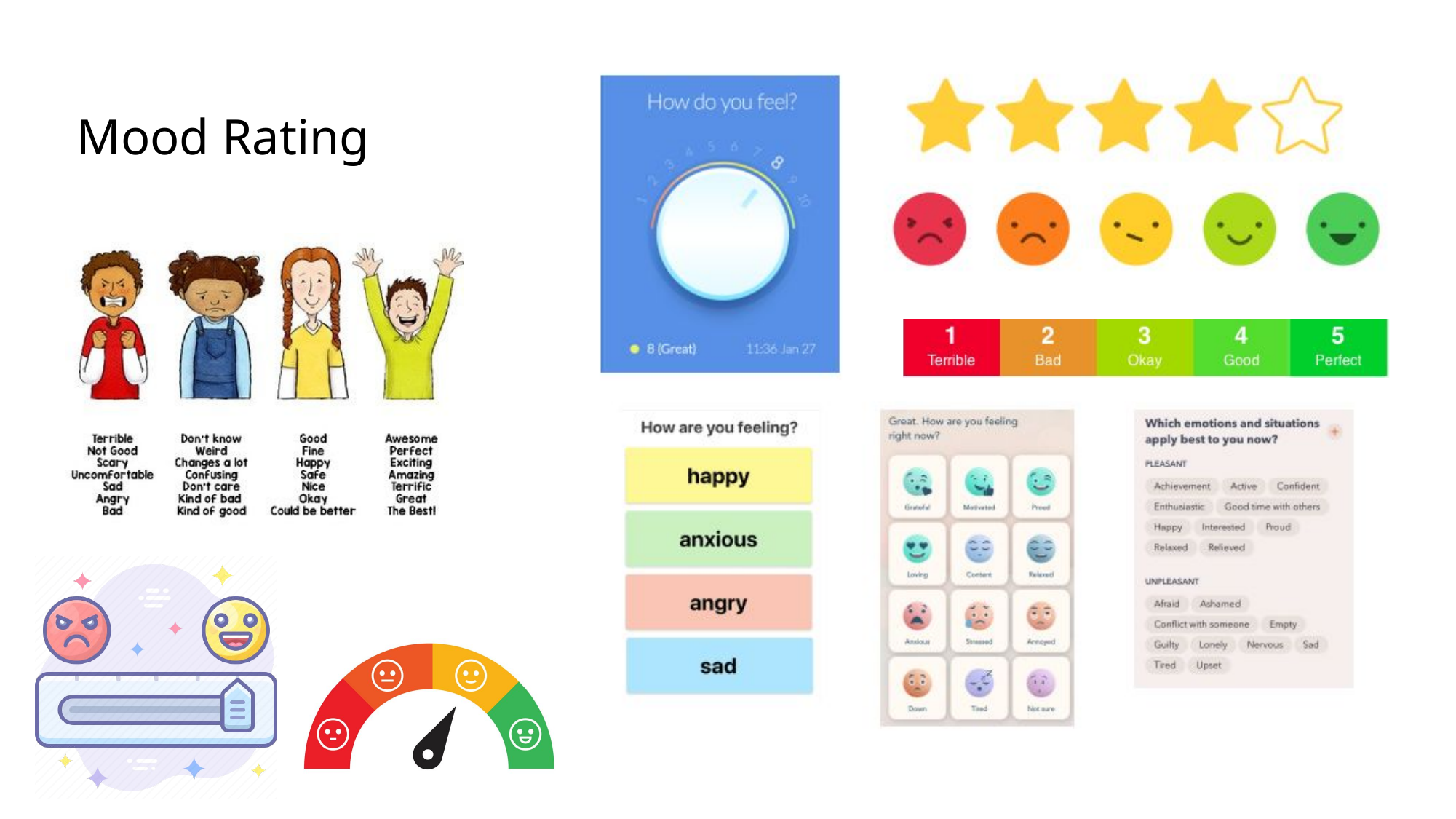

# Mood Rating

## Slide 4
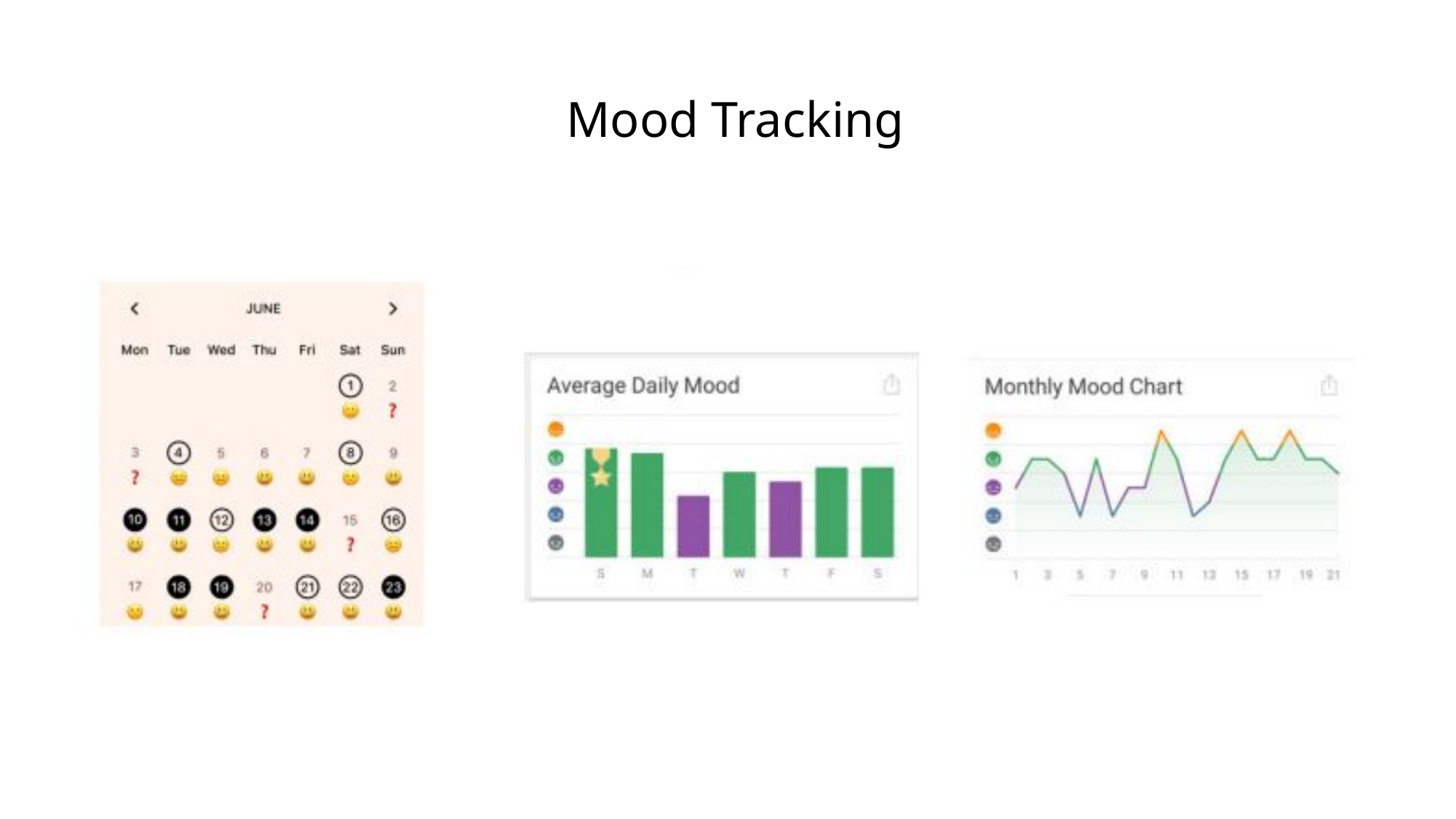

# Mood Tracking

## Slide 5
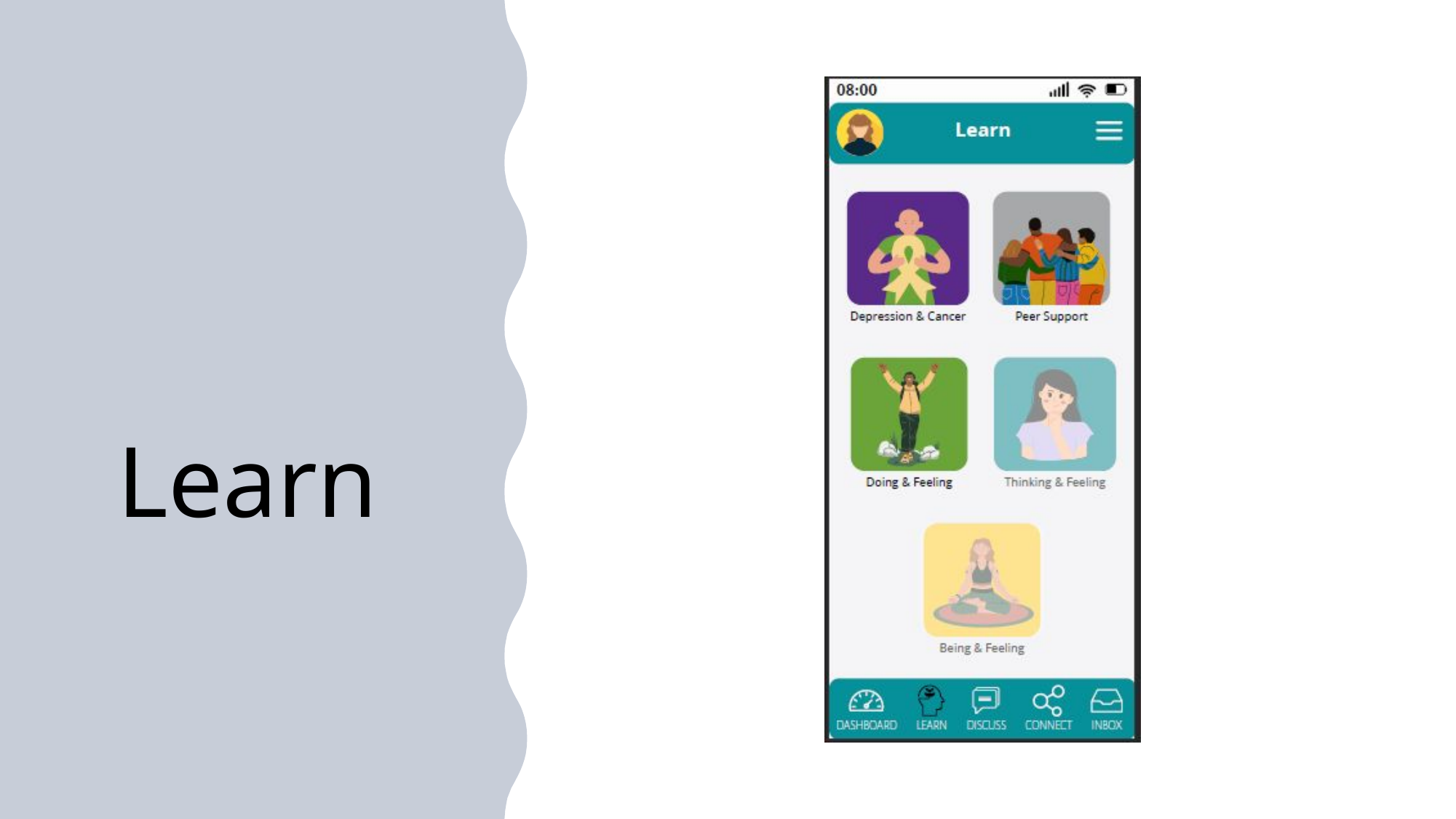

# Learn

## Slide 6
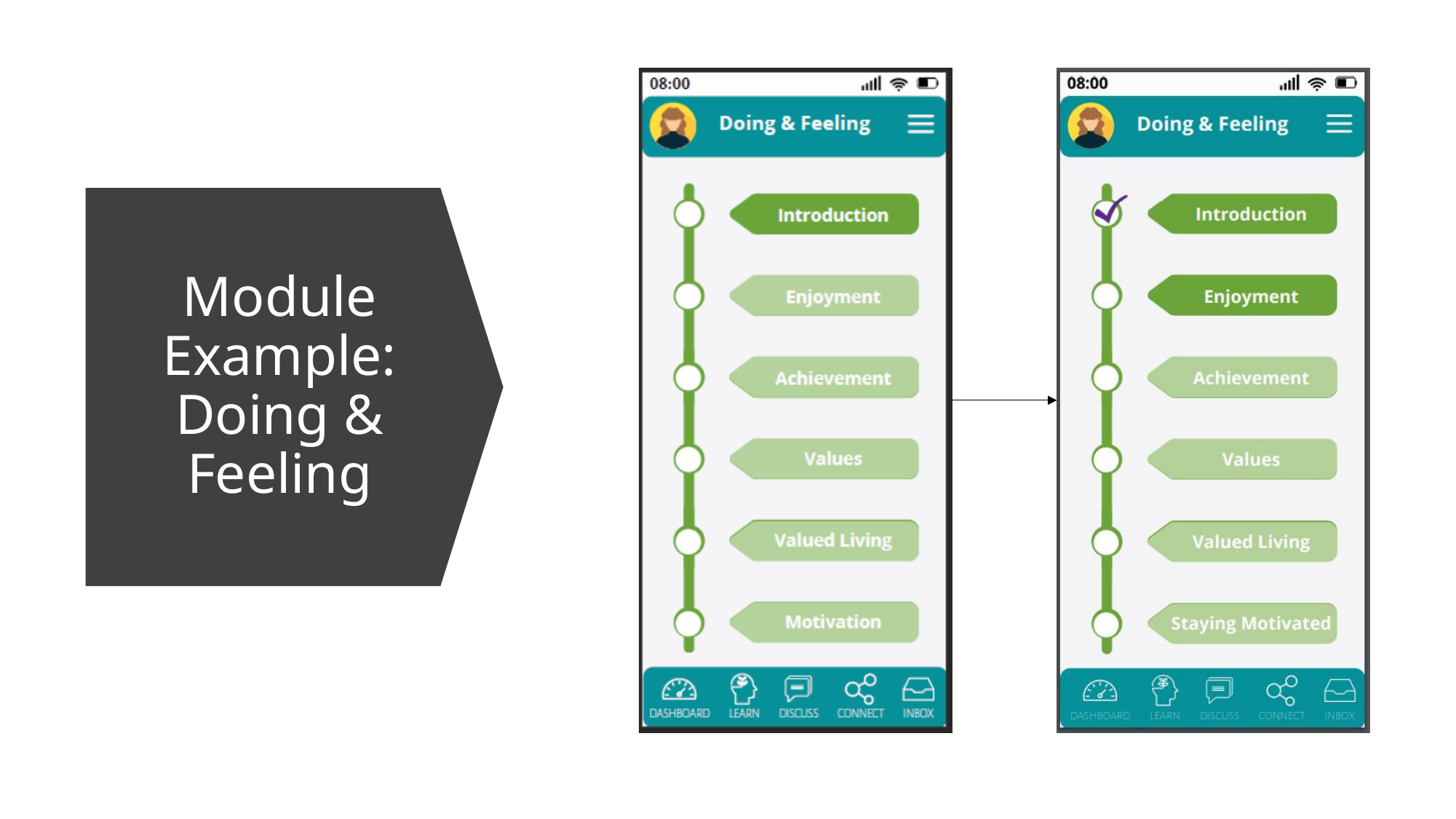

# Module Example: Doing & Feeling

## Slide 7
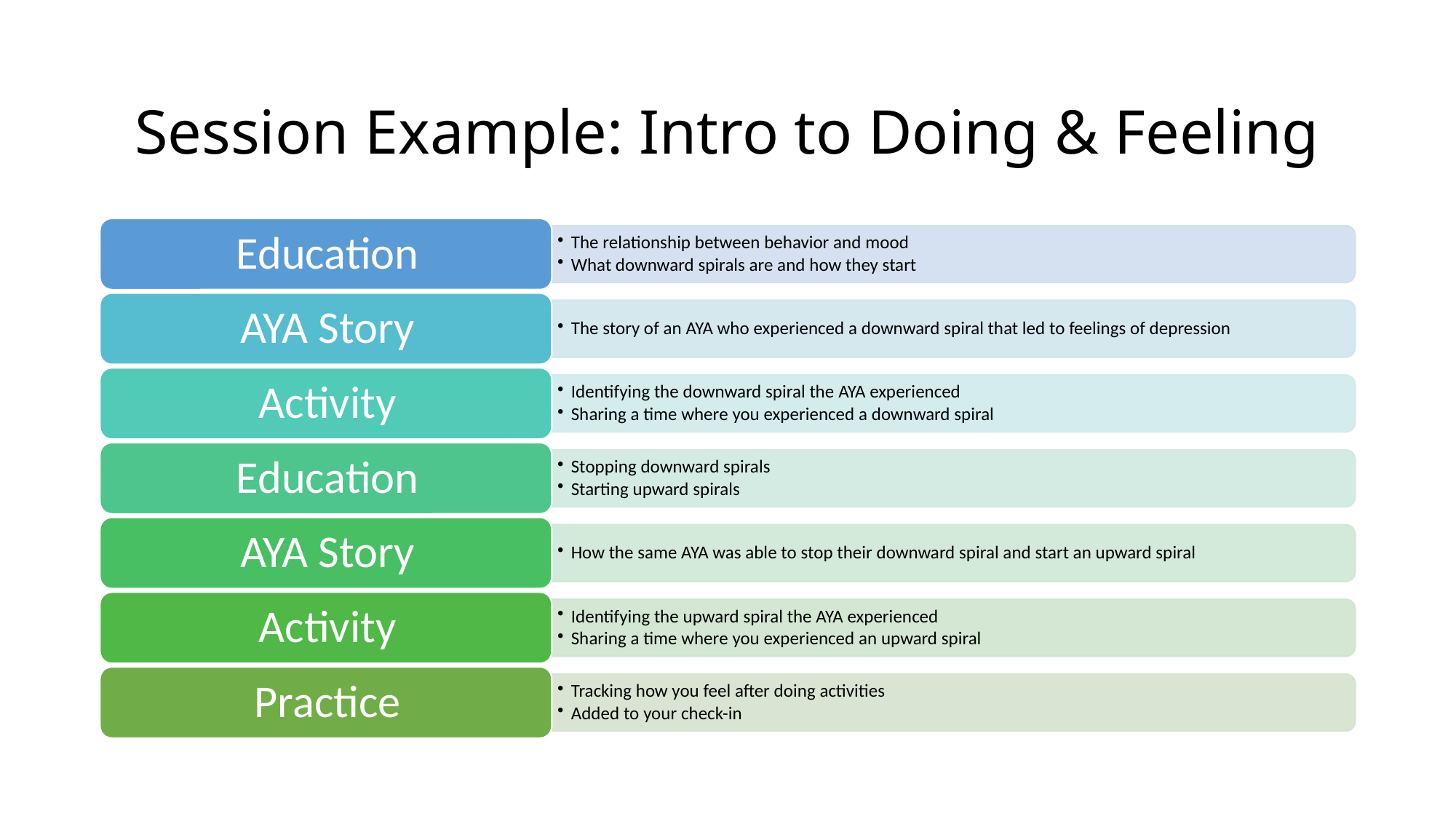

# Session Example: Intro to Doing & Feeling

## Slide 8
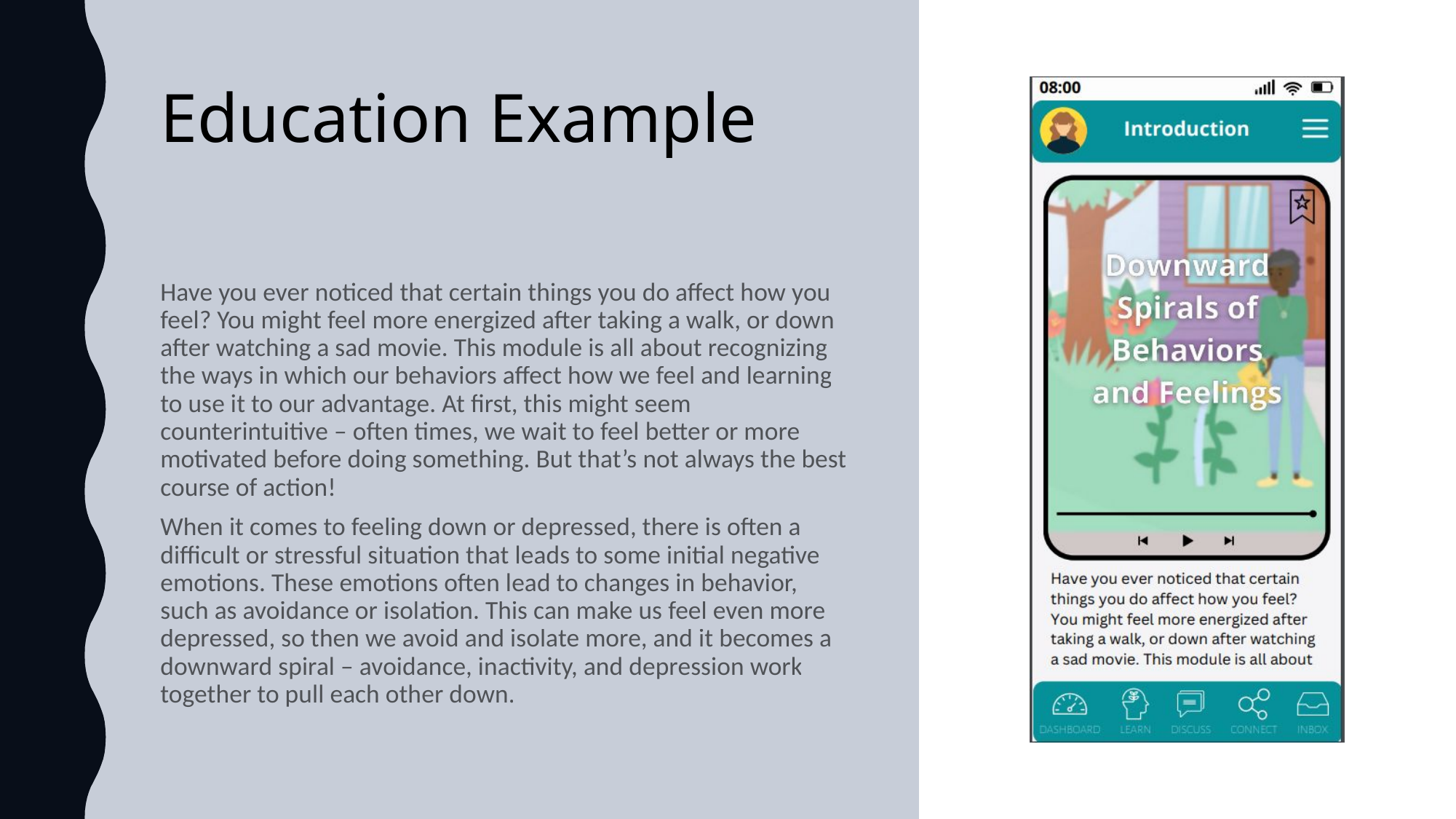

# Education Example
Have you ever noticed that certain things you do affect how you feel? You might feel more energized after taking a walk, or down after watching a sad movie. This module is all about recognizing the ways in which our behaviors affect how we feel and learning to use it to our advantage. At first, this might seem counterintuitive – often times, we wait to feel better or more motivated before doing something. But that’s not always the best course of action!
When it comes to feeling down or depressed, there is often a difficult or stressful situation that leads to some initial negative emotions. These emotions often lead to changes in behavior, such as avoidance or isolation. This can make us feel even more depressed, so then we avoid and isolate more, and it becomes a downward spiral – avoidance, inactivity, and depression work together to pull each other down.

## Slide 9
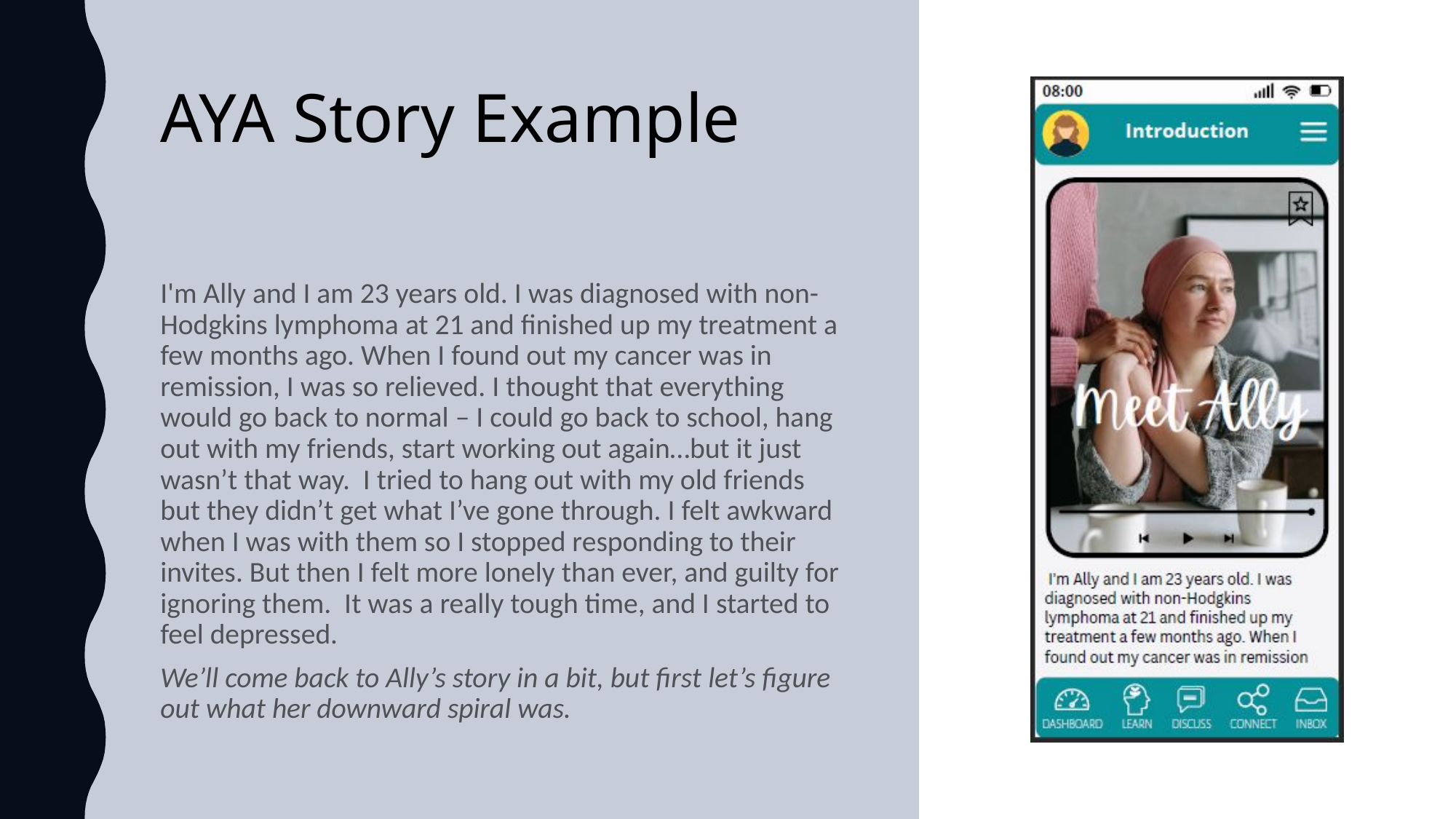

# AYA Story Example
I'm Ally and I am 23 years old. I was diagnosed with non-Hodgkins lymphoma at 21 and finished up my treatment a few months ago. When I found out my cancer was in remission, I was so relieved. I thought that everything would go back to normal – I could go back to school, hang out with my friends, start working out again…but it just wasn’t that way. I tried to hang out with my old friends but they didn’t get what I’ve gone through. I felt awkward when I was with them so I stopped responding to their invites. But then I felt more lonely than ever, and guilty for ignoring them. It was a really tough time, and I started to feel depressed.
We’ll come back to Ally’s story in a bit, but first let’s figure out what her downward spiral was.

## Slide 10
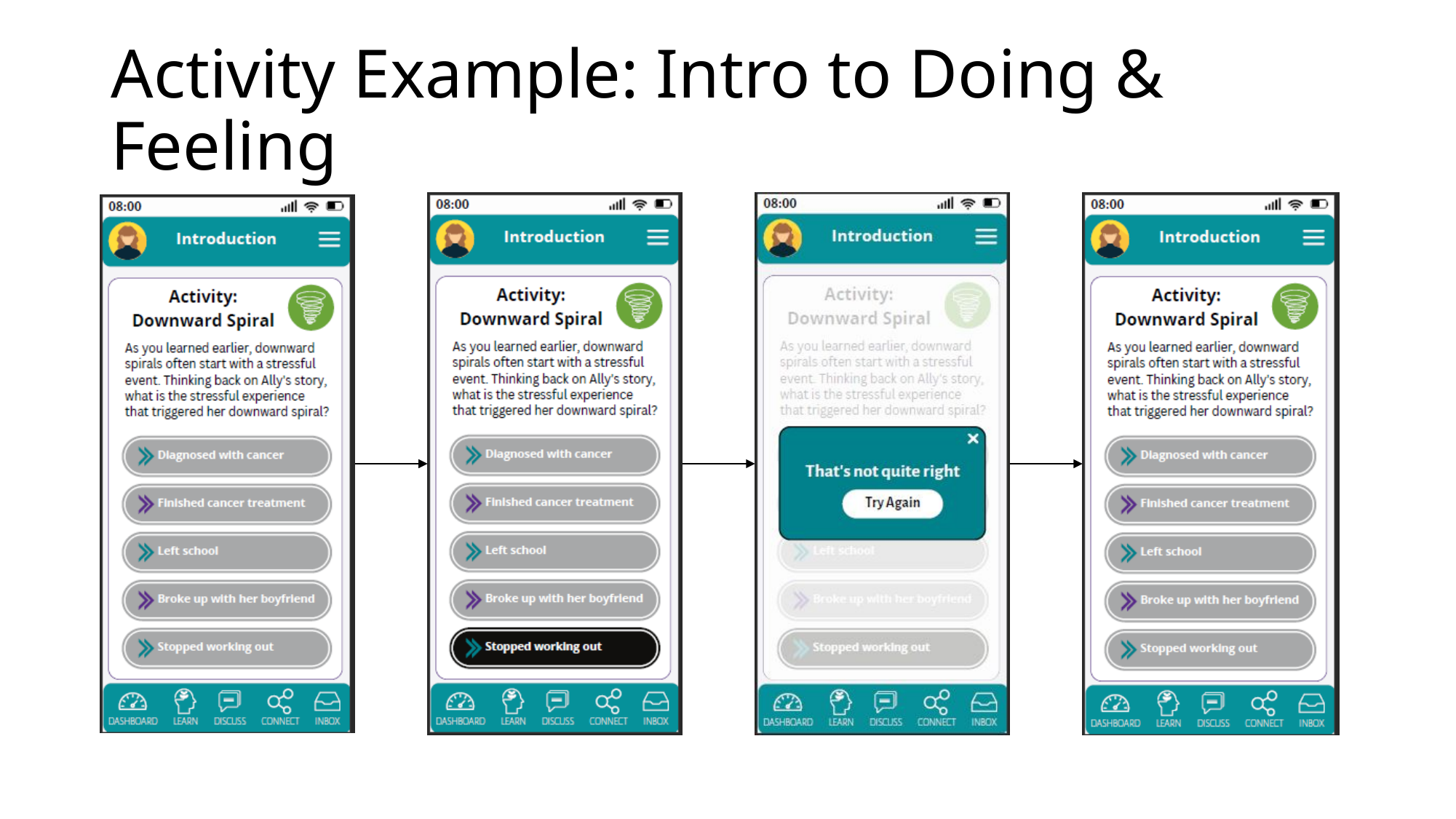

# Activity Example: Intro to Doing & Feeling

## Slide 11
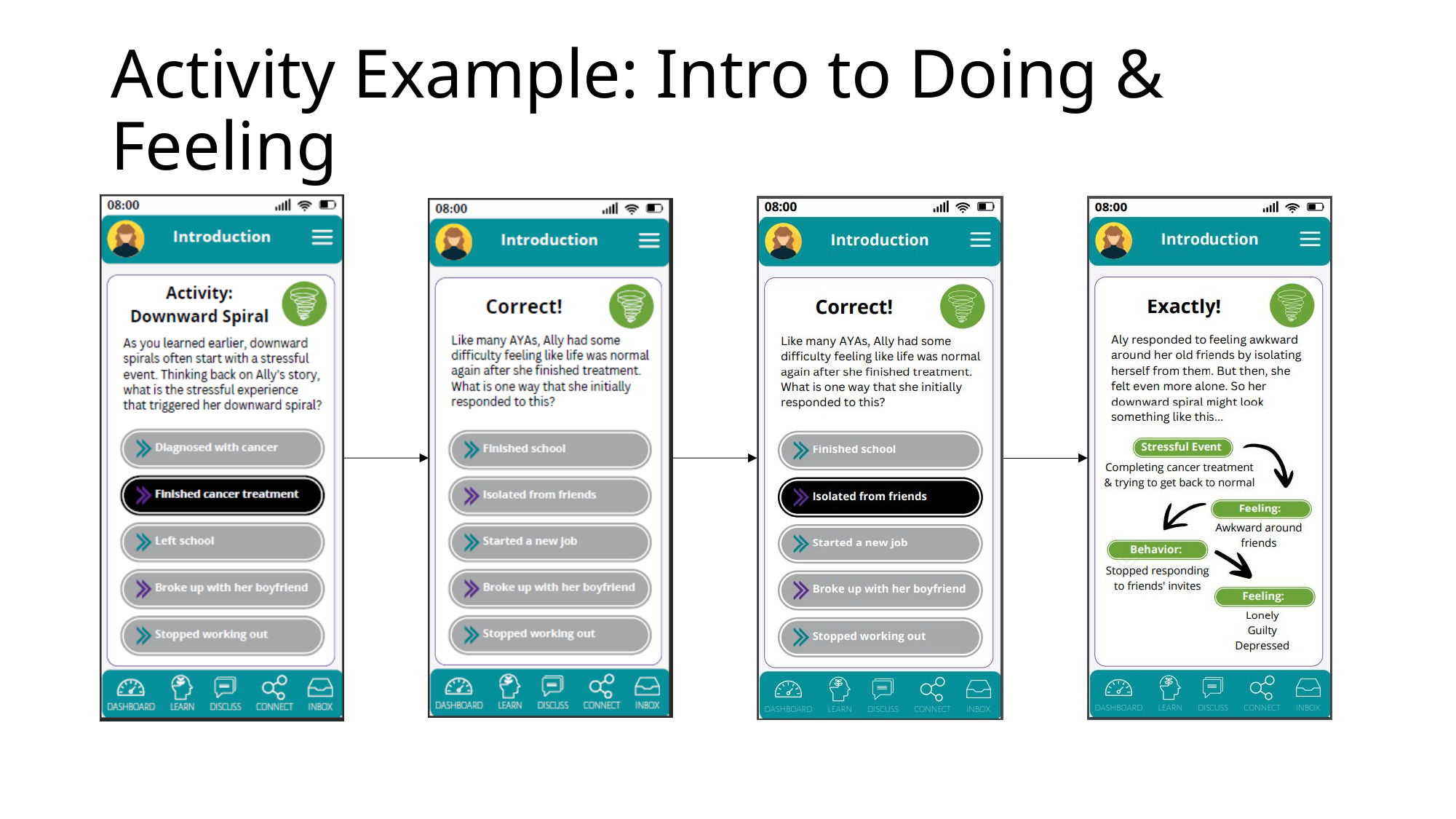

# Activity Example: Intro to Doing & Feeling

## Slide 12
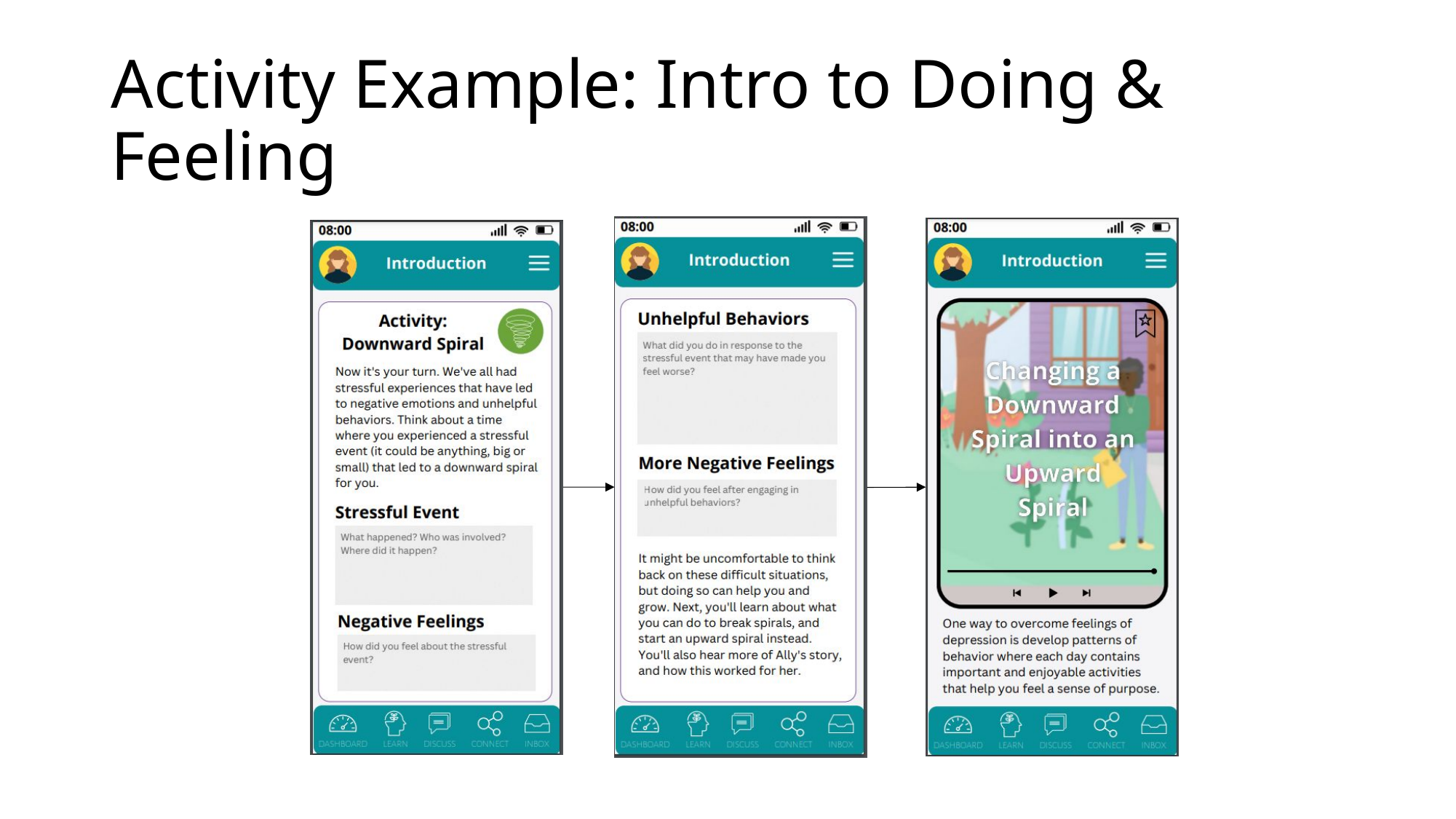

# Activity Example: Intro to Doing & Feeling

## Slide 13
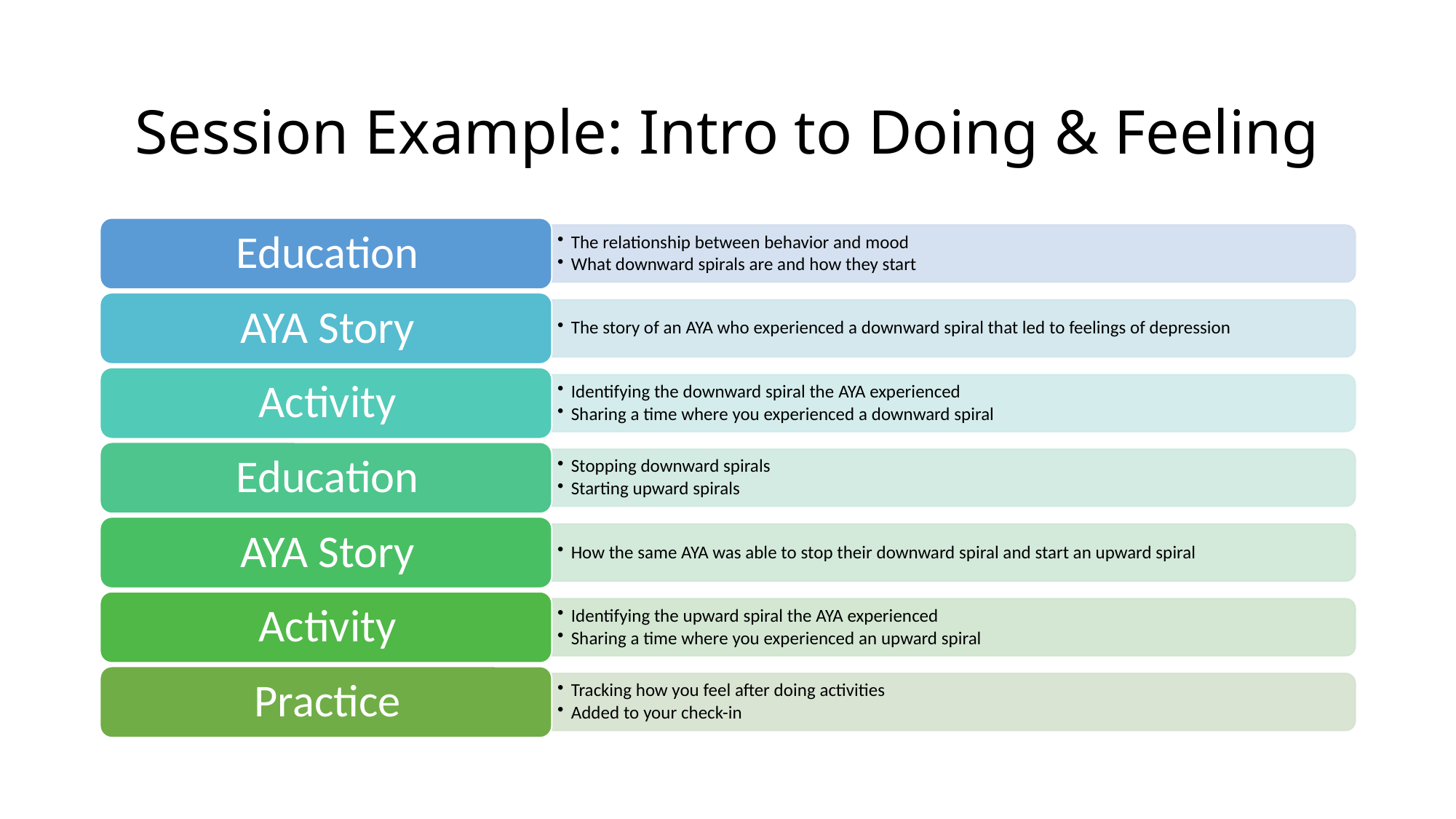

# Session Example: Intro to Doing & Feeling

## Slide 14
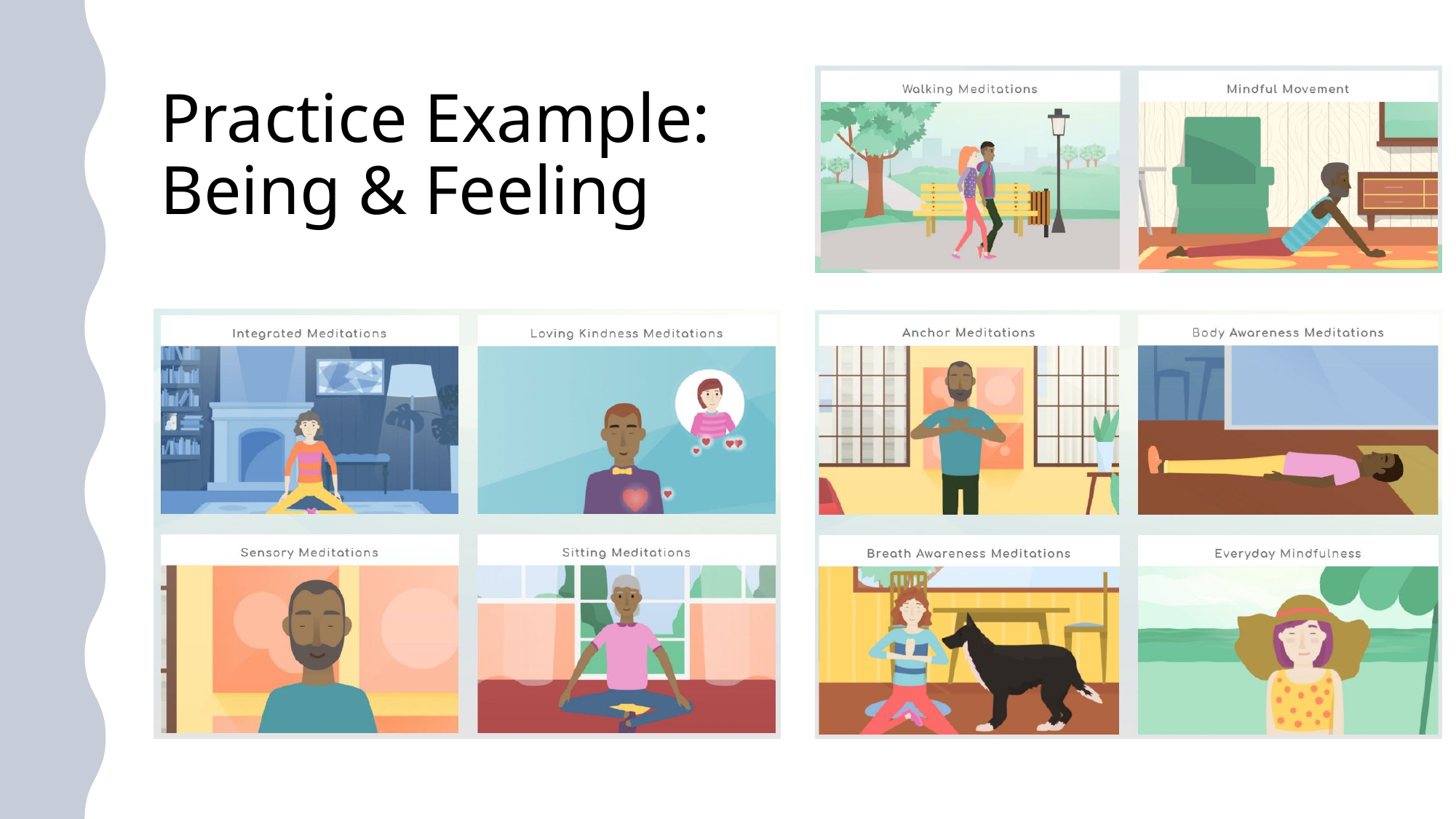

# Practice Example: Being & Feeling

## Slide 15
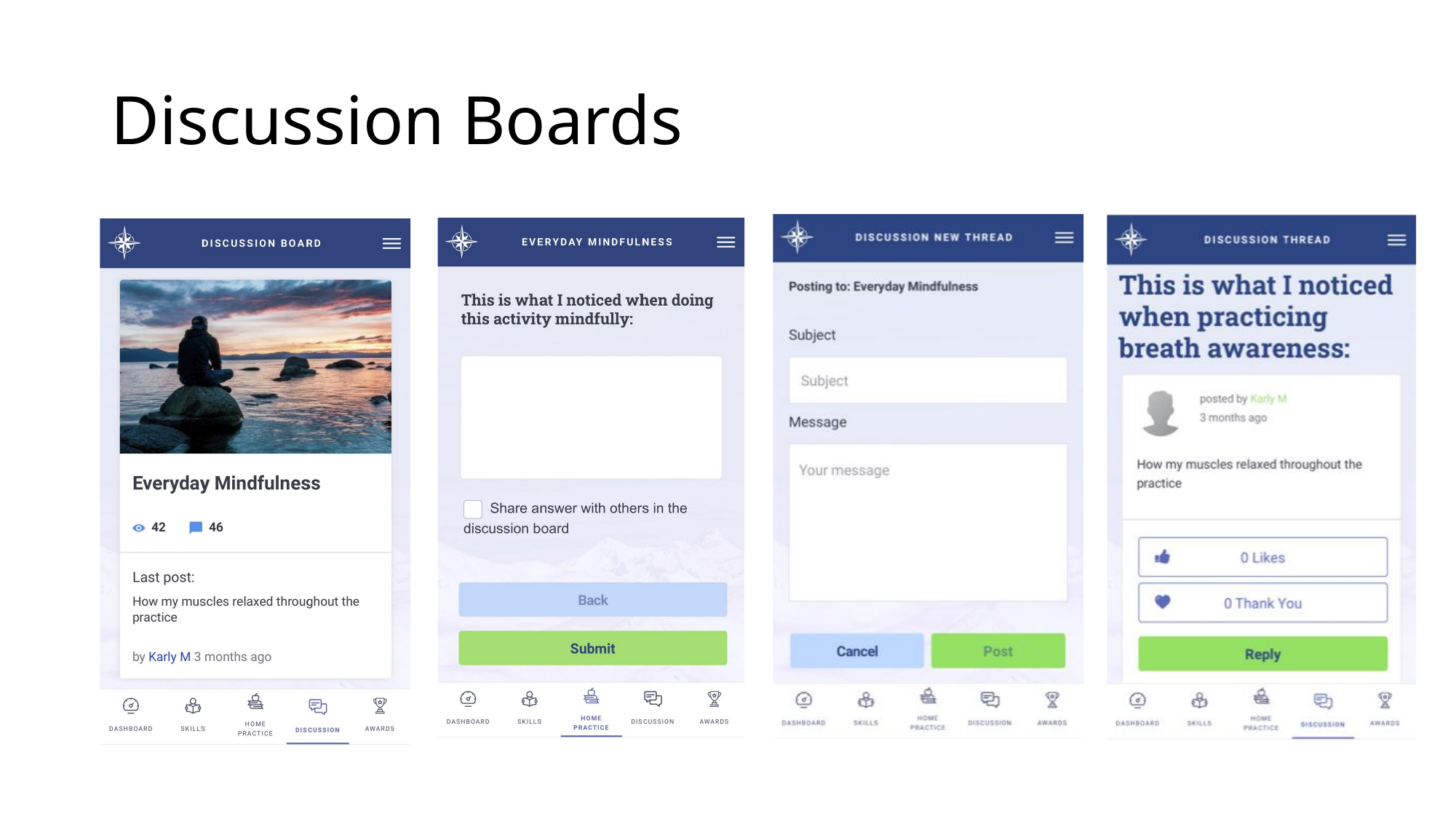

# Discussion Boards

## Slide 16
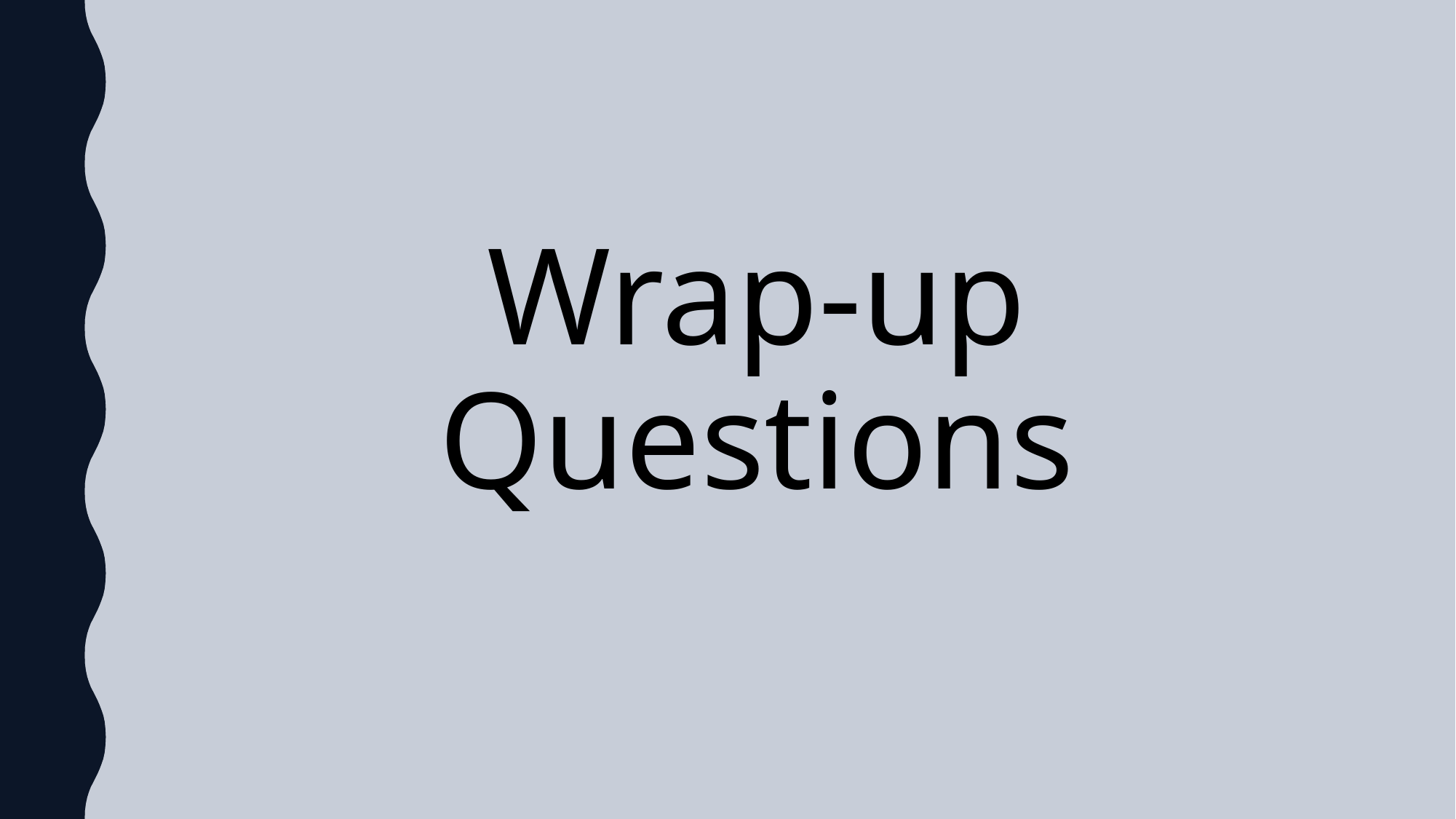

# Wrap-up Questions
